# The Typhoid Toxin Produced by the Nontyphoidal *Salmonella enterica* Serotype Javiana Is Required for Induction of a DNA Damage Response *In Vitro* and Systemic Spread *In Vivo*

**DOI:** 10.1128/mBio.00467-18

**Published:** 2018-03-27

**Authors:** Rachel A. Miller, Michael I. Betteken, Xiaodong Guo, Craig Altier, Gerald E. Duhamel, Martin Wiedmann

**Affiliations:** a Department of Food Science, Cornell University, Ithaca, New York, USA; b Department of Population Medicine and Diagnostic Sciences, Cornell University, Ithaca, New York, USA; c Department of Biomedical Sciences, Cornell University, Ithaca, New York, USA; University of Queensland

**Keywords:** DNA damage, *Salmonella*, nontyphoidal, typhoid toxin

## Abstract

The *Salmonella* cytolethal distending toxin (S-CDT), first described as the “typhoid toxin” in *Salmonella enterica* subsp. *enterica* serotype Typhi, induces DNA damage in eukaryotic cells. Recent studies have shown that more than 40 nontyphoidal *Salmonella* (NTS) serotypes carry genes that encode S-CDT, yet very little is known about the activity, function, and role of S-CDT in NTS. Here we show that deletion of genes encoding the binding subunit (*pltB*) and a bacteriophage muramidase predicted to play a role in toxin export (*ttsA*) does not abolish toxin activity in the S-CDT-positive NTS *Salmonella enterica* subsp. *enterica* serotype Javiana. However, *S.* Javiana strains harboring deletions of both *pltB* and its homolog *artB*, had a complete loss of S-CDT activity, suggesting that *S.* Javiana carries genes encoding two variants of the binding subunit. S-CDT-mediated DNA damage, as determined by phosphorylation of histone 2AX (H2AX), producing phosphorylated H2AX (γH2AX), was restricted to epithelial cells in S and G_2_/M phases of the cell cycle and did not result in apoptosis or cell death. Compared to mice infected with a Δ*cdtB* strain, mice infected with wild-type *S.* Javiana had significantly higher levels of *S.* Javiana in the liver, but not in the spleen, ileum, or cecum. Overall, we show that production of active S-CDT by NTS serotype *S.* Javiana requires different genes (*cdtB*, *pltA*, and either *pltB* or *artB*) for expression of biologically active toxin than those reported for S-CDT production by *S.* Typhi (*cdtB*, *pltA*, *pltB*, and *ttsA*). However, as in *S.* Typhi, NTS S-CDT influences the outcome of infection both *in vitro* and *in vivo*.

## IMPORTANCE

Nontyphoidal *Salmonella* (NTS) are a major cause of bacterial food-borne illness worldwide; however, our understanding of virulence mechanisms that determine the outcome and severity of nontyphoidal salmonellosis is incompletely understood. Here we show that S-CDT produced by NTS plays a significant role in the outcome of infection both *in vitro* and *in vivo*, highlighting S-CDT as an important virulence factor for nontyphoidal *Salmonella* serotypes. Our data also contribute novel information about the function of S-CDT, as S-CDT-mediated DNA damage occurs only during certain phases of the cell cycle, and the resulting damage does not induce cell death as assessed using a propidium iodide exclusion assay. Importantly, our data support that, despite having genetically similar S-CDT operons, NTS serotype *S.* Javiana has different genetic requirements than *S.* Typhi, for the production and export of active S-CDT.

## INTRODUCTION

Infections with nontyphoidal *Salmonella* (NTS) account for an estimated 93.8 million illnesses and 155,000 deaths per year globally (1), making NTS the third leading cause of bacterial food-borne disease worldwide (2). The *Salmonella* cytolethal distending toxin (S-CDT) (called the “typhoid toxin”) was first characterized in *Salmonella enterica* subsp. *enterica* serotype Typhi, the causative agent of typhoid fever (3, 4). However, recent studies have shown that S-CDT is not unique to *S.* Typhi, as >40 NTS serotypes are known to carry genes that encode S-CDT (5–7). Furthermore, *in vitro* characterizations have shown that these S-CDT-positive NTS serotypes produce active toxin (6, 8, 9).

S-CDT is an A_2_B_5_ toxin, composed of a pentameric ring of (i) PltB subunits which interact with host cell glycans (4, 10), (ii) an ADP-ribosyltransferase (PltA) with homology to the S1 subunit of the pertussis toxin (3, 4), and (iii) CdtB, which has nuclease activity (11). Infection with S-CDT-positive strains results in the accumulation of eukaryotic cells in the G_2_/M cell cycle phase (3, 6, 9, 12) and activation of the host cell’s DNA damage response (8). *In vivo*, administration of purified S-CDT from *S.* Typhi has been shown to partially recapitulate signs of typhoid fever in a mouse model (4, 10). S-CDT has also been shown to prolong carriage of *Salmonella* in 129S6/SvEvTac mice (13). Del Bel Belluz et al. showed that genetically engineered S-CDT-positive *S. enterica* subsp. *enterica* serotype Typhimurium (a naturally S-CDT-negative serotype [8]) persisted for a longer period of time *in vivo* than the S-CDT-negative parent strain did, which suggests that S-CDT alters the host-pathogen interaction *in vivo*, enabling S-CDT-positive *Salmonella* to persist in the host (13).

*S. enterica* subsp. *enterica* serotype Javiana is the fourth most commonly isolated NTS serotype causing infections in the United States (14) and is the most common S-CDT-positive NTS serotype (8). The majority of studies characterizing S-CDT activity have been performed using *Salmonella* serotype Typhi, which has several important genotypic and phenotypic differences from NTS serotypes (15–17). Furthermore, previous characterizations *in vitro* (3, 8, 9, 12, 18, 19), which were critical for asserting the activity of NTS S-CDT, were conducted using tumor-derived (cancerous) cell lines, which often have an altered DNA damage response (20). Use of a nontransformed cell line is therefore necessary to accurately reflect how infection with S-CDT-positive NTS impacts the cellular outcome of infection.

To better understand the mechanisms by which S-CDT-mediated intoxication occurs both *in vitro* and *in vivo*, we characterized the outcome of infection with S-CDT-positive *S.* Javiana at the cellular level, using a non-cancer-derived human intestinal epithelial cell line, and at the organismal level, by infecting C56BL/6 mice. We found that infection of human epithelial cells resulted in an altered cell cycle progression characterized by an accumulation of cells in the G_2_/M phase associated with DNA damage response activation in the absence of cell death. While *cdtB* and *pltA* were essential for toxin activity *in vitro*, *pltB*, *STY1887*, and *ttsA*, which are also carried within the S-CDT islet in *S.* Javiana, were not. Despite colonizing the ilea and ceca of infected mice at similar levels, infection with wild-type *S.* Javiana was associated with a higher bacterial load in the liver, suggesting that S-CDT might contribute to bacterial evasion of host innate inflammatory response mechanisms and allow for systemic infection with *S.* Javiana.

## RESULTS

### Infection of human epithelial cells with S-CDT-positive serotype *S.* Javiana induces a DNA damage response that significantly alters cell cycle progression.

**FIG 1:**
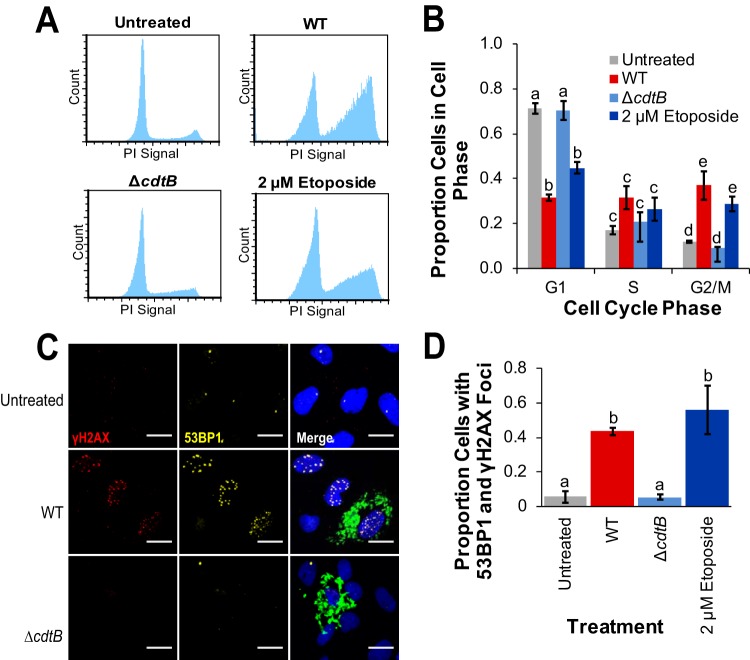
Infection with wild-type *S.* Javiana activates a DNA damage response and results in an accumulation of cells in the G_2_/M phase. HIEC-6 cells were infected with *S.* Javiana for 48 h prior to immunofluorescence staining or flow cytometry analyses. Cells treated with the topoisomerase inhibitor etoposide (at a final concentration of 2 µM) for 24 h served as a positive control. Data are from three independent experiments. (A) Representative histograms showing the cell cycle progression of HIEC-6 cells infected with wild-type (WT) and Δ*cdtB S.* Javiana strains; an untreated control is included to show a normal cell cycle progression, and cells treated with 2 µM etoposide demonstrate the accumulation of cells in G_2_/M phase due to DNA damage. (B) Quantification of cell cycle analyses shown in panel A. Histogram bars that do not share letters within a given cell cycle phase group (i.e., G_1_, S, and G_2_/M) are significantly different (*P* < 0.05). (C) Representative images of cells infected with *S.* Javiana strains (antibody stain shown in green) and positive controls (treated with 2 µM etoposide) and negative controls (untreated). The nuclei of HIEC-6 cells were stained with DAPI (blue). Scale bars represent 20 microns. (D) Quantification of the proportions of cells with at least four 53BP1 foci (shown in yellow) that colocalized with γH2AX foci (shown in red). Treatments that do not share letters have significantly different (*P* < 0.05) proportions of cells with 53BP1 foci that colocalized with γH2AX foci. *P* values were corrected for multiple comparisons using the Tukey honestly significant difference (HSD) correction method. Values in panels B and D are means ± standard errors of the means (error bars).

We previously established in a HeLa cell model that infection with S-CDT-positive NTS strains activates a DNA damage response and induces an accumulation of cells in the G_2_/M phase, while infection with either wild-type S-CDT-negative serotypes or Δ*cdtB* mutants does not do so (8). To overcome the limitations of using cell lines with altered DNA damage repair pathways, we infected normal (noncancerous) human intestinal epithelial cells (HIEC-6 cells [21]) with wild-type and Δ*cdtB* strains of *S.* Javiana to determine whether normal epithelial cells are also susceptible to S-CDT-mediated DNA damage. HIEC-6 cells infected with wild-type *S.* Javiana displayed a characteristic accumulation of cells in the G_2_/M phase of the cell cycle (average of 37% of the cells compared to 12% in uninfected controls [Fig. 1A and B]). Similar to uninfected controls, cells infected with the *S.* Javiana Δ*cdtB* strain had an average of 9% of cells in the G_2_/M phase, suggesting that the accumulation of cells in the G_2_/M phase is dependent on *cdtB* (Fig. 1A and B). Furthermore, HIEC-6 cells infected with wild-type *S.* Javiana also had a significantly lower proportion of cells in the G_1_ phase (Fig. 1B), compared to both uninfected controls (*P* = 0.0022) and cells infected with the Δ*cdtB* strain (*P* = 0.0008). Interestingly, the proportion of cells in S phase did not differ between Δ*cdtB* or wild-type strain-infected cells and uninfected controls (*P* > 0.05 for all comparisons). The altered cell cycle progression was consistent with induction of a DNA damage response, as infection with wild-type *S.* Javiana resulted in 43% of cells having at least four p53-binding protein 1 (53BP1) foci that colocalized with phosphorylated histone 2AX (γH2AX) foci (both DNA damage markers), compared to just 5% in uninfected control cells (*P* = 0.0113) or in cells infected with the Δ*cdtB* strain (*P* = 0.0213; Fig. 1C and D). Because active S-CDT is produced only by intracellular *Salmonella* (3, 8), we examined whether deletion of *cdtB* affected invasion efficiency as was reported previously for infection of HeLa cells with wild-type and Δ*cdtB S.* Javiana (9). Deletion of *cdtB* did not significantly affect invasion efficiency (*P* = 0.7113) in the Δ*cdtB* strain (0.59% versus 0.62% intracellular wild-type and Δ*cdtB S.* Javiana, respectively, normalized by CFU in the inoculum used for infection).

### *pltA* and *cdtB*, but not *pltB*, *ttsA*, or *STY1887* are required for expression of active S-CDT *in vitro*.

**FIG 2:**
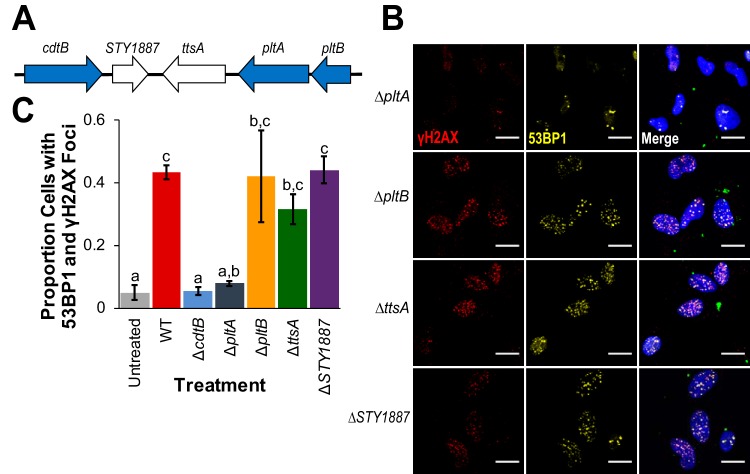
*pltB*, *ttsA*, and *STY1887* are not required for S-CDT-mediated DNA damage in HIEC-6 cells. HIEC-6 cells were infected with *S.* Javiana strains harboring single-gene deletions in the S-CDT islet. Immunofluorescence staining was performed for DNA damage response foci γH2AX and 53BP1. (A) Organization of the S-CDT islet in *S.* Javiana. Genes shown in blue represent genes encoding S-CDT subunits; genes shown in white are contained within the islet but do not encode protein products that constitute part of the S-CDT holotoxin. (B) Representative images of cells infected with wild-type *S.* Javiana and S-CDT single gene deletion strains (both colored green in the merged image); HIEC-6 cell nuclei are shown in blue. (C) Quantification of the proportions of cells with at least four 53BP1 foci that colocalized with γH2AX foci. Treatments that do not share letters have significantly different (*P* < 0.05) proportions of cells with 53BP1 foci that colocalized with γH2AX foci. Data represent three independent experiments. *P* values were corrected, to account for multiple testing, using Tukey’s HSD test. Error bars represent standard errors of the means.

S-CDT is encoded by genes in two adjacent operons (Fig. 2A) (3, 18). In addition to genes encoding S-CDT (*pltB*, *pltA*, and *cdtB*), the islet also carries genes encoding a putative bacteriophage muramidase (*ttsA*), which is hypothesized to play a role in toxin secretion (18), and *STY1887*, which has an unknown function in *S.* Typhi (18). To determine which genes in the S-CDT islet are essential for S-CDT-mediated DNA damage response activation in HIEC-6 cells infected with *S.* Javiana, we constructed strains with deletions of individual genes in the S-CDT islet.

Deletion of *cdtB* and *pltA* abolished the ability of *S.* Javiana to induce DNA damage response foci in HIEC-6 cells (Fig. 1C and D and Fig. 2B and C). HIEC-6 cells infected with the Δ*cdtB* strain or Δ*pltA* strain had an average of 5% and 8% of cells with at least four 53BP1 foci that colocalized with γH2AX foci, respectively, which did not differ significantly from uninfected controls (Δ*cdtB* strain versus control, *P* = 0.9835; Δ*pltA* strain versus control, *P* = 0.6469). In contrast, HIEC-6 cells infected with *STY1887* or *pltB* gene-deleted strains had an average of 44% and 42% of HIEC-6 cells with at least four 53BP1 and γH2AX foci, respectively, which did not differ from HIEC-6 cells infected with wild-type *S.* Javiana (*STY1887* versus wild-type, *P* > 0.9999; Δ*pltB* strain versus wild-type, *P* > 0.9999). Deletion of *ttsA* resulted in slightly lower levels of HIEC-6 cells with DNA damage response foci compared to wild-type *S.* Javiana (i.e., 32% of cells versus 43%), but this was not significantly different (*P* = 0.9795). Because *ttsA* was previously shown to be essential for S-CDT-mediated intoxication for *S.* Typhi (18), we aligned *ttsA* from typhoidal strains (i.e., *S*. Typhi strains CT-18 and Ty2 and *S*. Paratyphi A strain ATCC 11511) and nontyphoidal strains (e.g., *Salmonella* serotypes Javiana, Montevideo, and Schwarzengrund) to determine whether sequence diversity could partially explain the discrepant requirement for *ttsA* between *S.* Typhi and *S.* Javiana. Overall, *ttsA* was highly conserved (98.2% DNA sequence similarity) between typhoidal and nontyphoidal serotypes (see Fig. S1 in the supplemental material), with two nonsynonymous substitutions among 180 amino acids. Interestingly, these two amino acid substitutions were unique to the *S.* Montevideo *ttsA* sequence; the *ttsA* sequences from *S.* Javiana and the other serotypes (including both typhoidal and nontyphoidal serotypes) were identical, suggesting that sequence diversity in *ttsA* is unlikely to explain the lack of a *ttsA* requirement for production of active S-CDT in *S.* Javiana.

10.1128/mBio.00467-18.1 FIG S1  Amino acid alignment of TtsA from typhoidal and nontyphoidal serotypes. Amino acid sequences were predicted from *ttsA* sequences extracted from whole-genome sequence data. Strains included in analyses follow: *S.* Typhi strain CT-18, *S.* Typhi strain Ty2, *S.* Montevideo strain USDA-ARS-USMARC-1903, *S.* Paratyphi A strain ATCC 11511, *S.* Schwarzengrund strain CVM19633, and *S.* Javiana strain CFSAN0001992. Asterisks represent conserved amino acid residues. Amino acids highlighted in red represent amino acid changes present in TtsA from *S.* Montevideo compared to all other sequences of TtsA from all other serotypes. Download FIG S1, EPS file, 1.3 MB. Copyright © 2018 Miller et al. 2018 Miller et al. This content is distributed under the terms of the Creative Commons Attribution 4.0 International license.

### S. Javiana carries a *pltB* homolog, *artB*, which may substitute for PltB *in vitro*.

**FIG 3:**
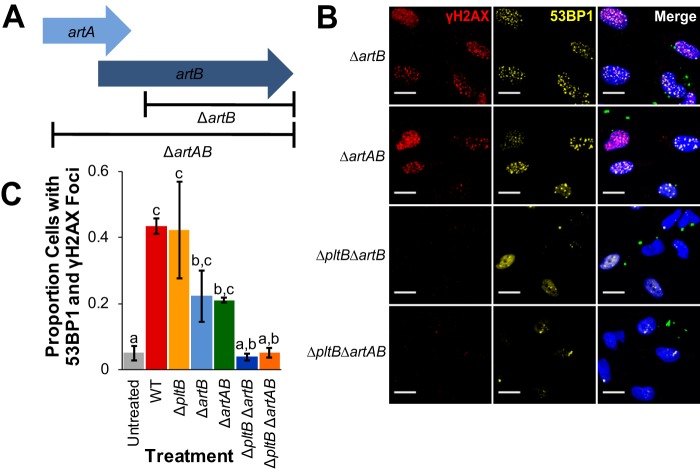
The presence of either *artB* or *pltB* is essential for S-CDT-mediated intoxication. Normal human intestinal epithelial HIEC-6 cells were infected with *S.* Javiana strains harboring either single gene deletions in *artB* or *artAB* or double deletions in *pltB* and either *artB* or *artAB*. At 48 hpi, cells were stained for the DNA damage response foci γH2AX and 53BP1. (A) Schematic of the gene deletions for Δ*artB* and Δ*artAB* strains. (B) Representative immunofluorescence images of HIEC-6 cells infected with *S.* Javiana deletion strains. *S.* Javiana cells (green) and HIEC-6 cell nuclei (blue) are shown. (C) Quantification of the proportions of cells with colocalized foci of at least four 53BP1 foci and γH2AX foci. Treatments that do not share letters are significantly different (*P* < 0.05). Results are from three independent experiments. *P* values were adjusted using Tukey’s HSD test to correct for multiple-comparison testing. Error bars represent standard errors of the means.

The S-CDT-negative (5, 8) *S. enterica* subsp. *enterica* serotype Typhimurium carries genes that encode an ADP-ribosylating toxin (called ArtAB) (22); these genes (*artA* and *artB*) are homologous to S-CDT subunits PltA and PltB (4–6). The ArtAB toxin is encoded by *artA* (encoding an ADP-ribosyltransferase) and *artB* (encoding the binding subunit) (22). Because deletion of *pltB*, encoding the binding subunit of S-CDT, did not have a significant effect on S-CDT-induced intoxication of infected HIEC-6 cells, we hypothesized that ArtB, which has homology to PltB (23), could potentially substitute for PltB *in vitro*. *S.* Javiana carries a truncated *artA* gene that overlaps with the full-length *artB* gene (Fig. 3A). To determine the importance of *artA* and *artB* in S-CDT intoxication, we constructed strains with deletions of (i) the entire *artAB* operon or (ii) just *artB* (Fig. 3A). HIEC-6 cells infected with Δ*artAB* or Δ*artB* strains had an average of 21% and 22% of cells with at least four 53BP1 foci that colocalized with γH2AX foci (Fig. 3B and C), respectively, which did not differ from HIEC-6 cells infected with either the wild-type or Δ*pltB* strain (*P* > 0.6 for all pairwise comparisons of wild-type, Δ*pltB*, Δ*artB*, and Δ*artAB* strains). However, infection with strains harboring deletions of both *pltB* and either *artB* or *artAB* abolished *S.* Javiana’s ability to induce DNA damage response foci in HIEC-6 cells (Fig. 3B and C). Compared to HIEC-6 cells infected with the Δ*pltB* strain, which had an average of 42% of cells with DNA damage response foci, infection with the Δ*pltB* Δ*artB* strain resulted in an average of 4% of cells with DNA damage response foci (*P* = 0.008; Fig. 3). Similarly, infection with the Δ*pltB* Δ*artAB* strain resulted in a significantly lower level of HIEC-6 cells with evidence of S-CDT intoxication (5% of cells with DNA damage response foci, *P* = 0.0198 compared to the Δ*pltB* strain).

**FIG 4:**
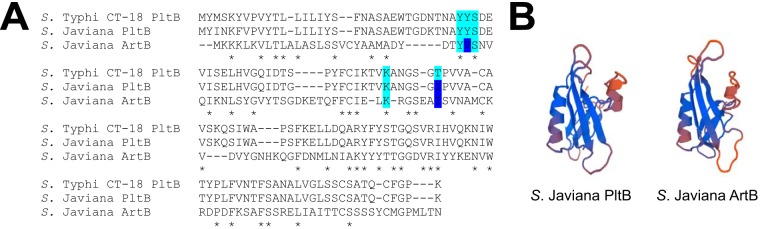
Key amino acid residues necessary for PltB binding to sugar moieties are also present in ArtB. (A) Alignments of translated amino acid sequences of PltB and ArtB from *S.* Javiana strain CFSAN0001992 and PltB from *S.* Typhi strain CT-18. Key residues that are conserved are shown on light blue background. Residues that differ between *S.* Typhi PltB and *S.* Javiana ArtB or PltB (24) are shown on dark blue background. Amino acid residues conserved in all three sequences (59) are indicated by an asterisk below the sequence alignment. (B) Predicted 3D structure of PltB and ArtB based on translated amino acid sequences of *pltB* and *artB* extracted from *S.* Javiana strain CFSAN0001992, generated using SWISS-MODEL online software (60).

To further explore whether ArtB could substitute for PltB as the binding subunit, we performed *in silico* comparisons of ArtB and PltB using an amino acid sequence alignment of ArtB with PltB and mapped the five amino acid residues that are predicted to interact with host cell receptors for toxin binding (24). Overall, three out of the five amino acid residues (Tyr33, Ser35, and Lys59), which have been predicted to make contact with host cell Neu5Ac-terminated glycans in *S.* Typhi (24), are conserved in ArtB from *S.* Javiana (Fig. 4A). While PltB from both *S.* Javiana and *S.* Typhi had Tyr34, *S.* Javiana ArtB had Val34. Interestingly, the Thr65 residue in *S.* Typhi PltB was mutated in both PltB and ArtB from *S.* Javiana; both ArtB and PltB contained isoleucine residues at this position. Finally, the predicted three-dimensional (3D) structures of the PltB and ArtB subunits from *S.* Javiana were structurally similar (Fig. 4B).

### *artB* is highly conserved among S-CDT-positive NTS serotypes.

To determine whether *artB* was conserved among S-CDT-positive serotypes, we aligned the predicted amino acid sequence of ArtB from *S.* Typhimurium DT104 (where it was first reported [22]) with those from other nontyphoidal and typhoidal S-CDT-positive serotypes. Predicted amino acid sequences of ArtB from S-CDT-positive serotypes clustered into three groups (Fig. S2). ArtB from nontyphoidal *Salmonella* serotypes Minnesota, Javiana, Rubislaw, Montevideo, and Schwarzengrund were highly similar, with just 2 out of 141 amino acids different (98.6% conserved among these serotypes). Similarly, predicted amino acid sequences for ArtB from *S*. Typhi CT-18 and *S*. Paratyphi ATCC 11511 strain had just one amino acid difference (99.3% identical among these serotypes). The predicted ArtB amino acid sequence from *S.* Typhimurium DT104 was distinct from the other sequences included in the alignment (73.2% identical to ArtB from *S*. Typhi CT-18).

10.1128/mBio.00467-18.2 FIG S2  Alignment of ArtB from typhoidal and nontyphoidal serotypes. (A) Phylogenetic tree of predicted ArtB amino acid sequences constructed using the James-Thorton-Thompson (JTT) model with 1,000 bootstrap repetitions in RAxML and rooted by midpoint. Bar represents 0.05 substitutions per amino acid residue. (B) Alignment of ArtB amino acid sequences. Asterisks represent amino acid residues that are conserved in all sequences in the alignment; amino acids highlighted in red represent amino acid substitutions compared to *S.* Typhimurium DT104 ArtB. Strains included in analyses follow: *S*. Typhimurium DT104, *S*. Javiana strain CFSAN0001992, *S*. Minnesota strain ATCC 49284, *S*. Montevideo strain USDA-ARS-USMARC-1921, *S.* Paratyphi A strain ATCC 11511, *S.* Rubislaw strain ATCC 10717, *S*. Schwarzengrund strain CVM19633, and *S.* Typhi strain CT18. Download FIG S2, EPS file, 1.5 MB. Copyright © 2018 Miller et al. 2018 Miller et al. This content is distributed under the terms of the Creative Commons Attribution 4.0 International license.

### S-CDT-mediated activation of a DNA damage response occurs primarily in the S and G_2_/M phases of the cell cycle in epithelial cells infected with wild-type *S.* Javiana.

**FIG 5:**
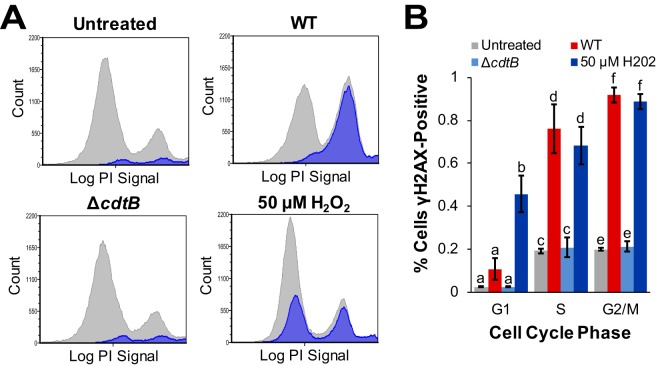
S-CDT-mediated DNA damage primarily occurs in the S and G_2_/M phases of the cell cycle of HIEC-6 human intestinal epithelial cells. HIEC-6 cells infected with *S.* Javiana wild-type (WT) and Δ*cdtB* strains for 48 h were stained to detect γH2AX and DNA content to determine the specific phases of the cell cycle during which S-CDT-mediated DNA damage occurred. (A) Representative histograms of DNA cell cycle analysis of HIEC-6 cells (gray) with γH2AX-positive cells overlaid (blue). (B) Quantification of γH2AX-positive HIEC-6 cells by cell cycle phase. Results are from two independent experiments. Treatments that do not share letters represent significantly different mean proportions (*P* < 0.05) of cells positive for a given cell cycle phase (e.g., proportions of γH2AX-positive cells in G_1_ were compared for the four treatment groups). *P* values were corrected for multiple comparisons using Tukey’s HSD test. Error bars represent standard errors of the means.

It is well established that the phase of the cell cycle in which DNA damage occurs will determine the DNA repair pathway that will be activated (25). Therefore, we infected HIEC-6 cells with wild-type and Δ*cdtB* strains of *S.* Javiana and used γH2AX staining to determine at which phase of the cell cycle S-CDT induced activation of a DNA damage response. Treatment with 50 µM H_2_O_2_, included as a positive control, consistently induced γH2AX activation (Fig. 5) throughout all phases of the cell cycle (46% to 89% cells γH2AX positive). HIEC-6 cells infected with wild-type *S.* Javiana had significantly higher proportions of γH2AX-positive cells only in the S phase (76% γH2AX-positive; *P* = 0.0175 compared to untreated controls) and G_2_/M phase (92% γH2AX-positive; *P* = 0.0044 compared to untreated controls) of the cell cycle. In the G_1_ phase, only 11% of cells infected with wild-type *S.* Javiana were γH2AX-positive, which was not significantly different from uninfected HIEC-6 cells (*P* = 0.1243) (Fig. 5). For a control, we also infected HIEC-6 cells with *S.* Javiana Δ*cdtB* strain, which did not result in higher mean proportions of γH2AX-positive cells compared to uninfected control cells; this result was consistent for all of the cell cycle phases (*P* > 0.999 for all comparisons with untreated cell populations).

### Infection with wild-type *S.* Javiana does not induce apoptosis or cell death.

**FIG 6:**
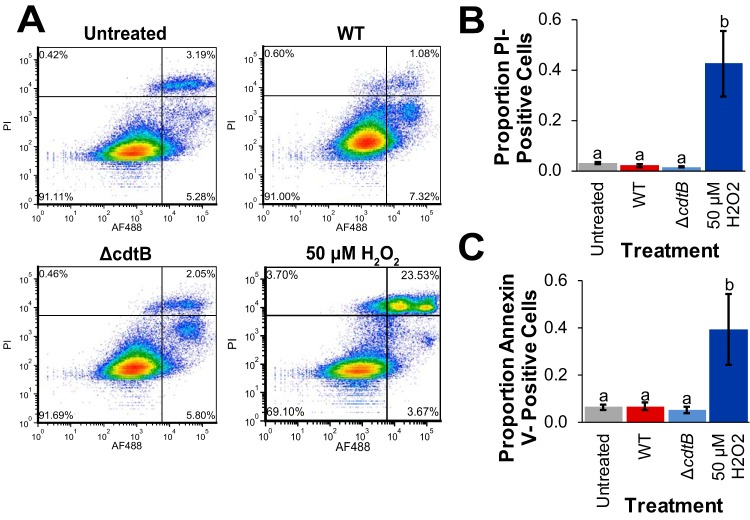
Infection of HIEC-6 human intestinal epithelial cells with *S.* Javiana does not induce apoptosis or cell death regardless of S-CDT status. HIEC-6 cells were infected with wild-type or Δ*cdtB S.* Javiana strains for 48 h or treated with 50 µM H_2_O_2_ for 8 h (for a positive control). Cells were stained with annexin V conjugated to Alexa Fluor 488 (AF488); PI was used as a live/dead stain. (A) Representative images of density plots of annexin V and PI staining of HIEC-6 cells infected with *S.* Javiana, treated with H_2_O_2_, or untreated. (B and C) Quantification of the proportions of PI-positive cells (B) and annexin V-positive cells (C) shown in panel A. Treatments that do not share letters have significantly different (*P* < 0.05) proportions of cells positive for either annexin V or PI staining. Results in panels B and C are from four independent experiments. Error bars represent standard errors of the means.

CDTs produced by several Gram-negative bacterial species have previously been shown to induce apoptosis in a variety of cell types (26–31). To determine whether infection with wild-type *S.* Javiana resulted in cell death (through apoptotic and other cell death pathways), we stained HIEC-6 cells infected with wild-type and Δ*cdtB S.* Javiana strains with annexin V to detect apoptotic cells and with propidium iodide (PI) to detect dead cells, regardless of the pathway leading to cell death (Fig. 6A). After 48 h, HIEC-6 cells infected with wild-type *S.* Javiana had an average of 7% cells positive for annexin V staining, which was not significantly different from the value for untreated control cells (10% annexin V-positive cells; Fig. 6B) but was slightly higher than the value for cells infected with the *S.* Javiana Δ*cdtB* strain (5% cells positive for annexin V). There was no significant difference in the mean proportion of annexin V-positive cells among untreated cells or cells infected with the wild-type or Δ*cdtB* strain (*P* > 0.6 for all comparisons). Because other, nonapoptotic cell death pathways exist (32), we also quantified the number of PI-positive cells (used as a live/dead stain). Cells treated with 50 µM H_2_O_2_, included as a positive control (Fig. 6A to C), had an average of 43% PI-positive cells (*P* = 0.0001 compared to untreated controls). The average proportion of PI-positive cells infected with the wild-type strain or the Δ*cdtB* strain (both had an average proportion of 2% PI-positive cells) did not differ significantly from uninfected controls (3%; *P* > 0.3 for all comparisons).

### Infection with wild-type *S.* Javiana results in higher bacterial counts in the liver, but not in the spleen, ileum, or cecum.

**FIG 7:**
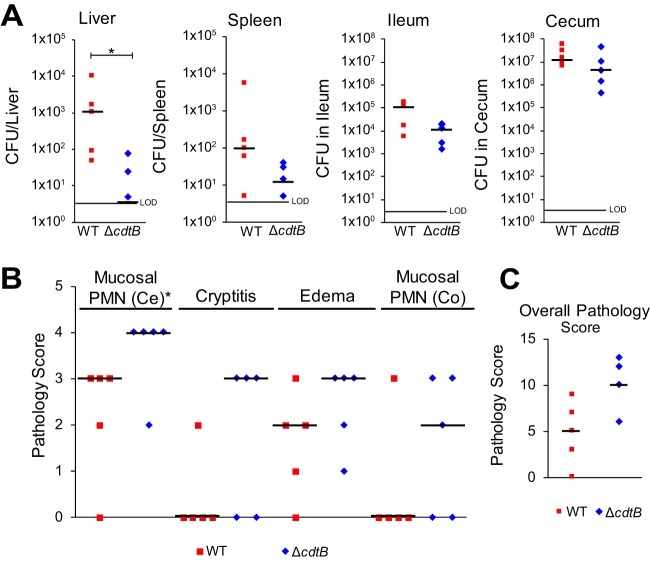
Infection of C56BL/6 mice with WT *S.* Javiana results in a higher bacterial load in the liver, but not in the spleen, ileum, or cecum, compared to infection with a Δ*cdtB* strain. Eight-week-old female C56BL/6 mice were orally inoculated with 1 × 10^9^ CFU *S.* Javiana wild-type or Δ*cdtB* strain (five mice in each group). At 48 hpi, the mice were euthanized, and tissues were harvested for bacteriologic and histological examination. (A) *S.* Javiana cells were enumerated from the liver, spleen, ileum, and cecum. The average bacterial load was calculated for each tissue. The limit of detection (LOD) for the average bacterial load for all tissues was 5 CFU. Differences in *S.* Javiana recovered from each tissue were compared using the nonparametric Kruskal-Wallis test. (B and C) Sections of the distal ileum, cecum, and proximal colon were fixed in buffered formaldehyde, embedded in paraffin, sectioned, and stained with hematoxylin and eosin. The levels of polymorphonuclear leukocytes (PMN) in the cecum (Ce) and proximal colon (Co) were scored on a scale of 0 to 4 (0 representing no PMNs and 4 representing large numbers of PMNs); cryptitis was assessed using the same scale. The degree of edema was scored from 0 (no edema) to 3 (severe edema). (C) The pathology score for typhlocolitis represents the sum of the scores (maximum score is 15) in panel B. Statistical differences in the counts of mice in each category of pathology score were assessed using the nonparametric Kruskal-Wallis test. Each symbol represents the value for an individual mouse. Black bars represent the median bacterial load (A) or median pathology scores (B and C) for mice infected with each *S.* Javiana strain. Values that are significantly different (*P* < 0.05) are indicated by a bar and asterisk. Only statistical associations with *P* < 0.05 are shown.

Because S-CDT is predicted to play an important role in the pathogenesis of typhoid fever (4), we infected C56BL/6 mice by pipetting 1 × 10^9^ CFU of wild-type and Δ*cdtB S.* Javiana strains into the oral cavity and then assessed the severity of infection 48 h postinfection. While mice infected with wild-type *S.* Javiana had significantly higher bacterial counts in the liver (*P* = 0.01597; median, 1,050 CFU/liver) than mice infected with Δ*cdtB S.* Javiana (median, 5 CFU/liver), the levels of *Salmonella* recovered from the spleen, ileum, and cecum did not differ significantly in mice infected with wild-type and Δ*cdtB* strains (*P* > 0.05 for pairwise comparisons of CFU recovered from the ileum and cecum; Fig. 7A).

Histological examination of the distal ilea, ceca, and proximal colons obtained from mice infected with the Δ*cdtB* strain revealed significantly higher cecal inflammation scores (*P* = 0.04863) compared to the scores for mice infected with wild-type *S.* Javiana (Fig. 7B). Mice infected with the Δ*cdtB* strain also had higher pathology scores for colonic cryptitis and mucosal polymorphonuclear leukocyte (PMN) infiltrate, and cecal submucosal edema, but these scores were not statistically significant (*P* > 0.05 for all comparisons between mice infected with wild-type and Δ*cdtB* strains). Overall, the typhlocolitis severity scores of mice infected with the Δ*cdtB* strain were higher but not significantly different (*P* = 0.07941) from the scores of mice infected with the wild-type *S.* Javiana (Fig. 7C).

## DISCUSSION

Although infections with S-CDT-positive NTS serotypes greatly outnumber infections with *S.* Typhi in the United States (14), molecular characterizations have primarily focused on S-CDT (typhoid toxin) produced by *S.* Typhi. In line with other genetic and phenotypic differences between nontyphoidal and typhoidal serotypes, and despite the fact that the serotypes carry genes encoding genetically similar variants of S-CDT, our data suggest that there are several differences in genes required for expression of biologically active S-CDT produced by *S.* Javiana, compared to what has been reported previously for the typhoid toxin produced by *S.* Typhi (3, 8, 18). We also show that S-CDT is an important virulence factor in *S.* Javiana, both *in vitro* and *in vivo*, as infection with wild-type *S.* Javiana induced a DNA damage response that resulted in an altered cell cycle progression of human intestinal epithelial cells *in vitro* and higher levels of *S.* Javiana translocation to the livers of infected mice.

### Inactivation of genes in the S-CDT islet for *S.* Javiana results in different phenotypes compared to S-CDT produced by *S.* Typhi.

We confirmed that *cdtB* and *pltA* are essential for S-CDT-mediated intoxication following infection with wild-type *S.* Javiana, which has been previously established for the S-CDT produced by *S.* Typhi (3). Interestingly, deletion of *pltB* and *ttsA* in *S.* Javiana did not abolish toxin activity (Fig. 2), despite the fact that these genes have previously been reported to be essential for toxin activity in *S.* Typhi (3, 18).

While deletion of *pltB* in *S.* Typhi is sufficient to abolish S-CDT-induced accumulation of cells in the G_2_/M phase (3), the *S.* Javiana Δ*pltB* strains, used in the present study, retained S-CDT activity *in vitro*, suggesting that PltB is not required for S-CDT expression by *S.* Javiana, consistent with a previous study by Mezal et al. (9). However, *S.* Javiana also contains a gene that encodes a *pltB* homolog (*artB*). Because deletion of both *pltB* and *artB* was required to abolish *S.* Javiana’s ability to activate a DNA damage response in HIEC-6 cells to a background level, it is likely that for *S.* Javiana, both ArtB and PltB may play a role in S-CDT-mediated DNA damage response induction *in vitro*. The conservation of key amino acid residues in ArtB, which have been shown to bind to host glycoproteins for PltB (4, 24), suggests that ArtB may possibly substitute for PltB as the binding subunit or may exist in the form of a heteropentameric binding subunit composed of both ArtB and PltB. Consistent with this hypothesis, recent data have confirmed that homopentamers of ArtB complexing with PltA and CdtB form an active S-CDT in *S.* Typhi (10). Furthermore, Gao et al. also showed that while PltB preferentially binds to Neu5Ac-terminated glycans (4, 10), ArtB binds both Neu5Ac, found on human cells, and Neu5Gc-terminated glycans, found on most other nonhuman mammalian cells; hence, inclusion of ArtB in the S-CdtB holotoxin could expand the range of host cells susceptible to S-CDT-mediated DNA damage response (10). Future investigations examining the presence of ArtB and PltB in the S-CDT holotoxin (including whether the holotoxin can represent a PltB-ArtB heteropentamer) will be important to understand the potential for ArtB to expand the range of hosts and cell types that can be targeted by S-CDT. Interestingly, *S.* Typhi strains CT-18 and Ty2 both carry the *artB* gene, and alignments of predicted amino acid sequences of ArtB for nontyphoidal and typhoidal serotypes show that ArtB is highly conserved (see Fig. S2 in the supplemental material) (6). Herdman et al. further confirmed that purified ArtB cloned from *S.* Typhi strain Ty2 binds to and induces vacuolization in a variety of host cell types, including Vero, CHO, and U937 cell lines (33).

Deletion of accessory genes *STY1887* and *ttsA* did not alter S-CDT activity *in vitro* in our study. In *S.* Typhi, *ttsA* was characterized as an *N*-acetyl-β-d-muramidase and was shown to be essential for exporting the toxin out of the host cell, but deletion of *STY1887* did not have any effect (18). Although a different cell type was used in that study (Henle cells versus HIEC-6 cells in our study), it is unlikely that this alone accounted for the observed differences, as both epithelial cell lines are of human origin. In their study, Hodak and Galan proposed that S-CDT release requires activity of the *N*-acetyl-β-d-muramidase (TtsA) and an unidentified holin, although attempts to show *in vitro* that purified *N*-acetyl-β-d-muramidase (TtsA) has muramidase activity were unsuccessful (18). Together, these results suggest that the S-CDT produced by nontyphoidal serotypes, such as *S.* Javiana, utilizes different mechanisms for S-CDT production, binding, and trafficking, compared to the S-CDT produced by *S.* Typhi. Alternatively, there could be other genes that compensate for the loss of *ttsA* function, as was observed for *pltB* and *artB*.

### Infection with wild-type *S.* Javiana does not induce apoptosis but does induce a DNA damage response that primarily occurs in the S and G_2_/M phases.

While the mechanism of S-CDT action and outcome of infection following exposure to S-CDT is poorly understood, our data suggest that DNA damage sustained following exposure to S-CDT occurs primarily in the S and G_2_/M phases of the cell cycle. Importantly, the cell cycle phase in which DNA damage is sustained determines which DNA repair pathway is activated (34). For example, in the G_1_ phase, homologous recombination is inhibited, and the error-prone nonhomologous end joining (NHEJ) DNA repair pathway is used to repair DNA double-strand breaks. In contrast, double-strand breaks sustained in the S or G_2_/M phase of the cell cycle are primarily repaired by homologous recombination (34, 35). Our data suggest that DNA damage resulting from infection with S-CDT-producing *S.* Javiana occurs during the S and G_2_/M phases of the cell cycle. This is also reflected by the characteristic accumulation of cells in the G_2_/M phase, which is the hallmark of CDT-induced DNA damage (36–38). Using a slightly different approach, Fedor et al. showed that *Escherichia coli* CDT induces DNA single-strand breaks that are converted to double-strand breaks in S phase (39). The authors also showed that homologous recombination repair proteins are associated with the double-strand breaks, suggesting that the DNA damage induced by CDT is likely repaired by homologous recombination (39). Although *E. coli* CDT is structurally different from S-CDT, conservation of CdtB, the active subunit, suggests that both toxins induce DNA damage via a similar mechanism (7, 37). Therefore, we hypothesize that the DNA damage induced by S-CDT from *S.* Javiana may be repaired in a manner similar to that of the DNA damage sustained following exposure to *E. coli* CDT, using the high-fidelity homologous recombination pathway rather than the error-prone NHEJ pathway. This would suggest a lower risk of introducing mutations after infection with S-CDT-positive NTS serotypes than would be expected if S-CDT-induced DNA damage were repaired by a more error-prone pathway, such as NHEJ.

### S-CDT contributes to *S.* Javiana systemic infection in C56BL/6 mice.

In contrast to what has been reported previously for S-CDT and other CDTs (19, 28, 40), we found normal human intestinal epithelial cells exposed to S-CDT did not undergo apoptotic cell death, suggesting that following exposure to S-CDT, epithelial cells either initiate a DNA damage response or enter senescence. While apoptosis is an anti-inflammatory cell death pathway (41), senescence is associated with the release of proinflammatory cytokines, known as the senescence-associated secretory phenotype (SASP), which serves as an innate signaling mechanism to recruit inflammatory cells to the site of senescent cells (42, 43). Consistent with our observations, Blazkova et al. showed that human epithelial cells exposed to *Haemophilus ducreyi* CDT enter a senescent state (44). Future work characterizing whether or not cells become senescent following S-CDT-mediated DNA damage will be necessary to understanding the potential contribution of SASP-associated inflammation in infections with S-CDT-positive NTS.

In mice, both the administration of purified S-CDT and infection with *S.* Typhimurium expressing *S.* Typhi S-CDT have been associated with suppression of the immune system (4, 13), which could enable S-CDT-producing NTS to evade host defense mechanisms (45). In our study, we found significantly higher levels of *S.* Javiana in the livers of mice infected with wild-type *S.* Javiana than in mice infected with the Δ*cdtB* strain; although the same trend was also observed for the spleen, differences in the levels of wild-type and Δ*cdtB S.* Javiana isolated from the spleen were not significant (*P* = 0.1127). Our data suggest that wild-type *S.* Javiana could use S-CDT to partially suppress the immune response, thereby allowing the *Salmonella* cells to spread to extraintestinal sites. In agreement with this, Song et al. (4) showed that mice injected with wild-type typhoid toxin (S-CDT) displayed a decrease in circulating monocytes, neutrophils, and lymphocytes five days after injection; mice injected with a catalytically inactive toxin had levels of white blood cells that were indistinguishable from those of untreated control mice (4). This suggests that S-CDT partially suppresses the innate immune response *in vivo*, which could enable S-CDT-positive *Salmonella* to evade immune cell killing and spread to extraintestinal sites. Furthermore, *H. ducreyi* CDT has been shown to induce apoptosis in human monocytic THP-1 cells (26). Although *Salmonella* resides primarily within Kupffer cells in the liver (46), it is possible that intoxication of these cells (i.e., exposure to DNA-damaging S-CDT) allows *Salmonella* to escape, and multiply outside Kupffer cells, in nearby hepatocytes. Importantly, mice infected with the Δ*cdtB* strain had higher cecal pathology scores than mice infected with the wild-type strain, despite similar *S.* Javiana colonization levels in the cecum. This suggests that S-CDT might alter the host innate inflammatory and immune responses to allow bacterial dissemination to extraintestinal sites. Although our study did not address the contribution of the adaptive immune response, Del Bel Belluz et al., using a chronic-infection mouse model, showed that *S.* Typhi S-CDT contributes to establishment of a persistent asymptomatic carrier status (13). We selected the streptomycin-treated mouse model to examine the effect of S-CDT on both gastroenteritis (47) and invasive disease. Streptomycin treatment has been shown to effectively alter the microbiome of mice (48), which enhances *Salmonella*’s ability to colonize the mouse intestine. Future studies using other mouse models, such as BALB/c mice, will be beneficial for confirming the results obtained in this study, as BALB/c mice are naturally susceptible to *Salmonella* infection (47). Furthermore, histological examination of liver and spleen samples will further expand our understanding of the mechanism by which S-CDT affects these tissues. While further studies will be helpful in understanding the role of S-CDT during an infection, our results suggest that S-CDT may play a key role in the acute phase of host infection with *S.* Javiana and contribute to systemic bacterial spread.

Despite an increasingly recognized role for CDTs in acute (4) and chronic (49, 50) disease, the role that S-CDT plays both in nontyphoidal and typhoidal salmonellosis is largely unknown. While our data suggest that a DNA damage response is induced when homologous recombination DNA repair pathways are active, future investigations determining the fate of the S-CDT-intoxicated cells *in vivo* remains an important gap in our current understanding of *S.* Javiana pathogenesis. Furthermore, understanding the regulation and *in vivo* targets of S-CDT will provide critical information concerning S-CDT contribution to the severity and outcome of salmonellosis. Whether S-CDT exposure predisposes individuals for chronic carriage or other conditions associated with the DNA-damaging effects of S-CDT, including genomic instability and certain cancers, is an area of active future investigation.

## MATERIALS AND METHODS

### Bacterial strains, epithelial cell line, and culture conditions.

*Salmonella* strains (Table 1) were preserved at −80°C in 15% (vol/vol) glycerol. Salmonella were grown on brain heart infusion (BHI) (Becton Dickinson, Sparks, MD) agar plates, which were incubated at 37°C. For *in vitro* infections, single colonies of *Salmonella* isolates grown on BHI agar plates were inoculated into 5-ml aliquots of LB (pH 8; 0.3 M NaCl), followed by incubation under static conditions at 37°C for 12 to 14 h. These cultures were subsequently subcultured 1:100 into fresh aliquots of LB (pH 8; 0.3 M NaCl), followed by incubation at 37°C under static conditions, until mid-log phase (optical density at 600 nm [OD_600_] of 0.4 to 0.5).

**TABLE 1:** *S.* Javiana strains used in this study

Strain	Relevant genotype
FSL S5-0395	Wild-type
FSL M8-0532	S5-0395 Δ*pltA*
FSL M8-0533	S5-0395 Δ*pltB*
FSL M8-0540	S5-0395 Δ*cdtB*
FSL M8-0577	S5-0395 Δ*ttsA*
FSL M8-0578	S5-0395 Δ*STY1887*
FSL M8-0582	S5-0395 Δ*artB*
FSL M8-0583	S5-0395 Δ*artAB*
FSL M8-0585	S5-0395 Δ*pltB* Δ*artB*::Kan^r^
FSL M8-0586	S5-0395 Δ*pltB* Δ*artAB*::Kan^r^
FSL M8-0590	S5-0395 Δ*cdtB* Δ*phoN*::Kan^r^
FSL M8-0591	S5-0395 Δ*phoN*::Kan^r^

Human intestinal epithelial cells (HIEC-6 cells; ATCC), derived from human fetal ileum (51), were grown in Opti-MEM medium supplemented with recombinant epidermal growth factor (10 ng/ml; Gibco-Invitrogen, Carlsbad, CA) and 10% (vol/vol) fetal bovine serum (FBS) (Gibco-Invitrogen) and were incubated at 37°C with 5% CO_2_. HIEC-6 cell supernatants were routinely tested for *Mycoplasma* infection using the VenorGEM *Mycoplasma* detection kit (Sigma-Aldrich, St. Louis, MO). For *Salmonella enterica* serotype Javiana infections, HIEC-6 cells were grown in 6-well or 24-well plates (Corning, Corning, NY). Cells were seeded into 6-well (2 × 10^5^ cells per well) or 24-well (1 × 10^5^ cells) plates 48 h (+4) h before infection.

### Strain construction.

Bacterial strains are shown in Table 1. The λ Red recombinase system was used to create all in-frame deletions (52). Briefly, *S.* Javiana FSL S5-0395 (wild type) containing the pKD46 plasmid (described in reference 52) was grown with shaking at 30°C in LB broth containing 100 μg/ml ampicillin and 0.01% l-arabinose (Sigma) to an OD_600_ of 0.5 to 0.7. Cells were transformed with kanamycin resistance cassettes containing flanking regions homologous to chromosomal sites of the gene targeted for deletion, using electroporation followed by a 2-h recovery in SOC medium (New England BioLabs [NEB], Ipswich, MA) at 37°C. PCR amplification was performed with the high-fidelity polymerase Q5 (NEB), used to amplify kanamycin cassettes from pKD46 with the primers listed in Table S1 in the supplemental material. Subsequently, cells were plated on LB supplemented with 50 μg/ml kanamycin and were incubated at 37°C for 18 to 24 h. Successful chromosomal integration of the kanamycin resistance cassette was confirmed by PCR. For removal of the kanamycin resistance cassette, cells were grown to mid-log phase (OD_600_, ~0.5 to 0.7) and were electroporated with pCP20, followed by incubation at 30°C for 2 h; colonies were selected by plating on LB agar, supplemented with 100 μg/ml ampicillin, for 20 to 24 h. For strains requiring multiple deletions, crosses with phage P22 were conducted (52). In-frame deletions were confirmed by Sanger sequencing. To generate the streptomycin-resistant strains for the mouse infection experiments, mid-log-phase wild-type *S.* Javiana was spread plated onto LB agar plates supplemented with 100 μg/ml streptomycin and incubated at 37°C. A single streptomycin-resistant colony was selected and was used to create the Δ*phoN*::Kan^r^ strain, so that both strains used for mouse infections (i.e., strains M8-0590 and M8-0591 [Table 1]) were generated from the same parental strain. The Δ*cdtB* Δ*phoN*::Kan^r^ strain was generated using transduction with P22 lysates from a Δ*cdtB* strain. Whole-genome sequencing was performed for both FSL M8-0590 and FSL M8-0591 strains and confirmed the integrity of the mutations generated, and the lack of single nucleotide polymorphisms (SNP); FSL M8-0591 and FSL M8-0590 showed a single SNP difference, located in a noncoding region.

10.1128/mBio.00467-18.4 TABLE S1  Primers used in this study. Download TABLE S1, DOCX file, 0.02 MB. Copyright © 2018 Miller et al. 2018 Miller et al. This content is distributed under the terms of the Creative Commons Attribution 4.0 International license.

### *Salmonella* infection of HIEC-6 cells.

Treatments (i.e., strain used, negative- or positive-control treatments) were randomly assigned to HIEC-6 cells seeded in 6-well or 24-well plates. HIEC-6 cells were infected with approximately 2 × 10^6^ CFU or 4 × 10^6^ CFU *Salmonella* for HIEC-6 cells seeded in 24-well and 6-well plates, respectively. After incubation of the infected cells for 1 h at 37°C (with 5% CO_2_), HIEC-6 cells were washed three times with phosphate-buffered saline (PBS), followed by incubation with medium supplemented with 100 μg/ml gentamicin (Gibco) for 1 h at 37°C to kill extracellular bacteria. Subsequently, HIEC-6 cells were washed an additional three times with PBS and were then maintained in medium containing 10 μg/ml gentamicin (Gibco) to prevent recurrent infection and bacterial outgrowth during incubation.

### Flow cytometry for DNA content.

Cell cycle analyses were performed on populations of HIEC-6 cells that were infected with *Salmonella* strains as described above. Briefly, HIEC-6 cells were washed once with PBS and harvested using 0.25% trypsin-EDTA (Gibco). Cells were fixed in ice-cold 70% ethanol and stored at −20°C. Ethanol-fixed cells were permeabilized with PBS containing 0.1% Tween 20 (PBS-T) and bovine serum albumin (BSA) (1 g/100 ml) (Sigma-Aldrich) at room temperature for 10 min. Cells were subsequently stained (10 min at room temperature) with a solution containing propidium iodide (PI) (Thermo Fisher Scientific, Waltham, MA) at a final concentration of 50 μg/ml and RNase A (Sigma-Aldrich) at a final concentration of 100 μg/ml. Stained cells were held at 4°C for no longer than 4 h, prior to DNA content analysis using the BD FACSARIA sorter. Cells were gated to exclude doublets and multiplets (Fig. S3), as described previously (53).

10.1128/mBio.00467-18.3 FIG S3  Gating strategies used in this study. Download FIG S3, EPS file, 1.6 MB. Copyright © 2018 Miller et al. 2018 Miller et al. This content is distributed under the terms of the Creative Commons Attribution 4.0 International license.

### Immunofluorescence staining.

Immunofluorescence staining for γH2AX and 53BP1 foci was performed at 48 (+2) h postinfection (hpi) as described previously (8). Briefly, HIEC-6 cells grown on 12-mm coverslips (Thermo Fisher Scientific) were washed with PBS and were then fixed with 4% formaldehyde in PBS at room temperature for 10 to 15 min. Fixed cells were permeabilized with 0.1% Triton X-100 in PBS at room temperature for 10 min. Permeabilized cells were blocked with 10% (vol/vol) normal (healthy) donkey serum (Sigma) in PBS containing 0.1% Tween 20 (PBS-T) (J. T. Baker, Phillipsburg, NJ) for 1 h at room temperature. Incubation with primary and secondary antibodies was performed for 1 h at room temperature using the following dilution factors in PBS-T: polyclonal goat anti-*Salmonella* antibody (KPL antibodies; KPL, Gaithersburg, MA; 1:500), mouse anti-γH2AX (EMD Millipore, Billerica, MA; 1:500), rabbit anti-53BP1 (Novus Biologicals, Littleton, CO; 1:500). Incubation with secondary antibodies diluted 1:200, except where noted, in PBS-T was performed for 1 h at room temperature: donkey anti-goat conjugated to Alexa Fluor 488, donkey anti-rabbit conjugated to Alexa Fluor 555 (diluted 1:500), and donkey anti-mouse conjugated to Alexa Fluor 647 (all Thermo Fisher Scientific). Nuclei were stained with 4′,6-diamidino-2-phenylindole (DAPI) (Thermo Fisher Scientific) at a final concentration of 1 μg/ml for 5 min at room temperature. Slides were mounted onto microscope slides with glycergel (Dako, Carpinteria, CA) and imaged using a Zeiss 710 confocal microscope. Images were processed with FIJI software, and cells were counted using the cell counter plug-in (54). At least 50 nuclei were analyzed per slide to identify cells that had at least four 53BP1-positive foci that colocalized with γH2AX foci. The observer was blind to treatments while collecting and analyzing images.

### Apoptosis staining.

HIEC-6 cells were infected with *Salmonella* as described above. Hydrogen peroxide was added as a positive control (final concentration of approximately 50 μM) to uninfected control cells approximately 8 h prior to staining. Detached cells (potentially dead cells) were included in analyses by collecting supernatants of treated HIEC-6 cells via centrifugation at 3,000 rpm for 5 min. The adherent cells were trypsinized with 0.25% trypsin-EDTA (Gibco), washed with PBS (4°C), and subsequently combined with cells collected from supernatants via centrifugation at 3,000 rpm for 5 min. Cells were stained for 15 min at room temperature with annexin V conjugated to Alexa Fluor 488 (Invitrogen) and PI. Cells were analyzed within 2 h of staining using the BD FACSARIA sorter. Refer to Fig. S3 for a description of the gating strategy used.

### γH2AX flow cytometry.

At 48 (+2) h postinfection, *Salmonella*-infected HIEC-6 cells and controls were washed once with PBS and subsequently harvested with 0.25% trypsin-EDTA. The positive-control cells were incubated with H_2_O_2_ at a final concentration of 200 μM for 1 h at 37°C. Cells were fixed with 4% formaldehyde (Thermo Fisher Scientific) at room temperature for 5 min and permeabilized with 0.1% Triton X-100 for 10 min at room temperature. Cells were then blocked with 3% BSA (Sigma-Aldrich) in PBS for 30 min. Antibody staining was performed at room temperature for 1 h with mouse anti-γH2AX antibody (Millipore) and for 30 min with donkey anti-mouse conjugated to Alexa Fluor 488 (Thermo Fisher Scientific); both antibodies were used at a 1:200 dilution. Labeled cells were subsequently stained for cell cycle analysis by incubation with PI for 10 min at room temperature (using 300 µl of the PI staining solution described above). Cells were analyzed using the BD FACSAria sorter within 2 h of staining. The gating strategy used is shown in Fig. S3.

### Mouse infection experiments.

Ten female C57BL/6 mice were purchased from the Jackson Laboratory (Bar Harbor, ME). All experimental procedures were approved by the Cornell University Institutional Use and Care of Animals Committee. Mice were administered 20 mg streptomycin sulfate by pipetting 50 μl of a 400-mg/ml stock solution into the oral cavity 24 h prior to infection with *Salmonella*. Strains FSL M8-0590 and FSL M8-0591 (Table 1) were inoculated into 25 ml of LB broth and grown with aeration (200 rpm) at 37°C for 18 h. These bacterial cultures were pelleted, resuspended in PBS, and concentrated to achieve 1 × 10^10^ CFU in 500 μl of PBS. Mice were orally inoculated by pipetting 50 µl of this inoculum (containing 1 × 10^9^ CFU *Salmonella*; five mice in each group) into the oral cavity of each mouse. Mice were euthanized 48 h postinfection, and the liver, spleen, ileum, and cecum were aseptically removed at necropsy. The liver and spleen were homogenized and resuspended in 1 ml of PBS. The distal ileum and cecum were transferred to a 50-ml Falcon tube containing 5 ml of PBS followed by vortex agitation for 5 min (i.e., wash 1). A second wash was performed by transferring the organs to a new 50-ml Falcon tube containing 5 ml of PBS, and the tubes were vortexed for an additional 5 min (i.e., wash 2). The organs were removed, and the two corresponding PBS wash solutions were combined. Homogenates were serially diluted and spread plated on LB supplemented with 50 µg/ml kanamycin. The plates were incubated for 20 to 24 h at 37°C. Homogenates were stored at 4°C and replated if the bacterial counts were below detectable levels.

### Histological analysis.

The distal ileum (approximately 0.5 cm), cecum, proximal colon (approximately 1.5 cm), and associated mesenteric lymph nodes were harvested, fixed in 10% neutral buffered formalin, embedded in paraffin, sectioned at 5-µm thickness, and stained with hematoxylin and eosin for histological assessment; liver and spleen samples were not collected for histological analysis. Sections of the cecum and proximal colon were scored by a board-certified veterinary pathologist, in a blind fashion, for the presence and distribution of polymorphonuclear leukocytes within (i) the lamina propria of the intestinal mucosa and (ii) inside intestinal crypts (cryptitis) using a modified version of a previously described (48) scale of 0 to 4 (0, none; 1, rare; 2, few scattered; 3, many groups; 4, large numbers). Additionally, the degree of edema within the submucosa of intestinal sections was scored on a scale of 0 to 3 (0, none; 1, mild; 2, moderate; 3, severe). Typhlocolitis severity was calculated as the sum of the scores for the categorical parameters of the cecum and proximal colon (maximum of 15).

### Data analysis.

All raw data are available by request. For codes used in the statistical analyses, refer to Data Set S1. Statistical analyses were performed using R version 3.4.2. with packages lme4 1.1-14 (55), lmerTest (56) version 2.0-33, and lsmeans version 2.27-2 (57).

10.1128/mBio.00467-18.5 Data Set S1  Codes used in the statistical analyses. Download Data Set S1, PDF file, 0.6 MB. Copyright © 2018 Miller et al. 2018 Miller et al. This content is distributed under the terms of the Creative Commons Attribution 4.0 International license.

For comparing the proportions of (i) cells in a given cell cycle phase (Fig. 1B), (ii) cells with at least four 53BP1foci colocalized with γH2AX foci (Fig. 1D, 2C, and 3C), (iii) cells positive for γH2AX staining by cell cycle phase (Fig. 5B), and (iv) cells positive for annexin V (Fig. 6B) or PI (Fig. 6C) staining, proportions were first logit transformed, and linear mixed effect models were fit (see Data Set S1 for complete model details and output). Pairwise comparisons to determine differences in cellular outcomes dependent on the strain used for infection (e.g., wild-type versus Δ*cdtB* strain) were performed using the lsmeans package. *P* values were corrected for multiple comparisons using the false-discovery rate correction in the stats package (58).

For comparing bacterial loads recovered from the spleen and liver and the fecal contents of the ileum and cecum, the nonparametric Kruskal-Wallis test was used. Data that were below the detectable levels were plotted on graphs as being at the limit of detection (LOD) (e.g., 5 CFU in the liver and spleen) and were included as levels at the LOD in statistical analyses. Due to the low variability in the organ weights (see Data Set S1), we compared the overall bacterial load, rather than correcting by organ weight.

Statistical differences in pathology scores were assessed using the Kruskal-Wallis test to determine whether (i) cecal mucosal PMN, (ii) cecal cryptitis, (iii) cecal edema, and (iv) colonic mucosal PMN scores were significantly different between mice infected with wild-type and Δ*cdtB* strains. Comparison of differences in the mean total pathology scores between mice in the groups inoculated with wild-type and Δ*cdtB* strains was done using the Kruskal-Wallis test.

For comparing the rates of invasion/adherence between wild-type and Δ*cdtB* strains, a linear mixed-effect model was fit using logit transformed CFU/milliliter counts, and lsmeans was used to determine whether the mean CFU recovered were statistically different between strains.
