# Supplementary material for: The Typhoid Toxin Produced by the Nontyphoidal Salmonella enterica Serotype Javiana Is Required for Induction of a DNA Damage Response In Vitro and Systemic Spread In Vivo
Source: mBio. 2018 Mar 27;9(2):e00467-18. doi: 10.1128/mBio.00467-18 (PMC5874915; doi:10.1128/mBio.00467-18)
Supplement: TABLE S1 [file mbo002183808st1.docx]

**Table S1. Primers used in this study**

| Primer Name | Purpose | Primer sequence (5' to 3') |
| --- | --- | --- |
| RM91cdtBKanup | Deletion of *cdtB* | AACACATATATCATTCAGATAAAAAAGTAATAATCGGGAGAGTAGATATCGTGTAGGCTGGAGCTGCTTC |
| RM92cdtBKandown | Deletion of cdtB | TATTCTGCACCTTACGCTCAAAGTACATGTCGTCAACGCTATTTACTCACATATGAATATCCTCCTTAG |
| RM97cdtBup_F | Confirm deletion of *cdtB* | CAACGTCATGAAACAATGGGTTATG |
| RM96cdtBdown_R | Confirm deletion of cdtB | ATATTCTGCACCTTACGCTCAAAGTAC |
| RM174PcdtBGFP_EcoRI_F | Complementation of *cdtB* | TAAGCAGAATTCTAAGTCACCTGTTTTGTGTTGAG |
| RM199_artBupstreamF | Confirm deletion of artB | CTTCCGCCGTCTGTTGCTTA |
| RM200_artBdownstreamR | Confirm deletion of artB | GGTGACAGTATCTGCGGCATTGTT |
| RM209_STY1887kanF | Deletion of *STY1887* | AATTATGAGTTGTTTTACCAGTCCAGCAATTATGGTGTAGGCTGGAGCTGCTTC |
| RM210_STY1887kanR | Deletion of *STY1887* | TTAATTATGTTGTATGGTATGAGAATGATAGTATTTCATATGAATATCCTCCTTAG |
| RM211_STY1887F | Confirm deletion of STY1887 | GTACTTTGAGCGTAAGGTGCA |
| RM212_STY1887R | Confirm deletion of STY1887 | TGGCGGTTTGTGATGATAGCATA |
| RM213_ttsAkanF | Deletion of *ttsA* | TTACAACCTTACCCGTTCCTTTATCCATCCGTGTAGGCTGGAGCTGCTTC |
| RM214_ttsAkanR | Deletion of *ttsA* | ATCTTTGCAGCTATCCTTAGTAGAGAAGGTGGTTACCATATGAATATCCTCCTTAG |
| RM215_ttsAF | Confirm deletion of *ttsA* | GTGGCTTTCACCAGTTCTCT |
| RM216_ttsAR | Confirm deletion of *ttsA* | GATGCAAGACCTGTAATAGAACTT |
| RM221_artABkanF | Deletion of *artAB* | ATGCAGTTACAAAGTGAGTATGTATCTGTAGTGTAGGCTGGAGCTGCTTC |
| RM222_cdtBcomp_AatII_R | Complementation of *cdtB* | GACGTCCATGTCGTCAACGCTATTTACTCA |
| RM223_artBintF | Screen for *artB* | GCTCTTGCGTCATTATCCAGTGTT |
| RM224_artBintR | Screen for artB | CACTTTACACATGGCATTGACACTAA |
| RM225_artB2kanF | Deletion of *artB* | AGTAATGTTCAGATTAAAAACCTGTCTTATGGTGTGTAGGCTGGAGCTGCTTC |
| RM226_artB2kanR | Deletion of *artB* | CCTGTTCTTTAGAGCGTTTAATACCCAGCAACATCATATGAATATCCTCCTTAG |
