# Supplementary material for: The Typhoid Toxin Produced by the Nontyphoidal Salmonella enterica Serotype Javiana Is Required for Induction of a DNA Damage Response In Vitro and Systemic Spread In Vivo
Source: mBio. 2018 Mar 27;9(2):e00467-18. doi: 10.1128/mBio.00467-18 (PMC5874915; doi:10.1128/mBio.00467-18)
Supplement: Data Set S1 [file mbo002183808sd1.pdf]

```
> #### Statistical Analyses for S-CDT Manuscript
> library(lme4)
> library(lmerTest)
> library(lsmeans)
>
> ###DATA DIRECTORY
> setwd("Z:/ram524_Rachel Miller/2016-2017_HIEC-6 cell model S-CDT project
/Statistical Analyses")
>
> ###FIGURE 1B: LMER MODEL FOR PROPORTION OF CELLS IN G1/S/G2-M
> cc1=read.csv("F1_FC.csv", sep=",", strip.white = TRUE, header=TRUE)
>   ###G2
>   m_g2=lmer(G2Mlogit~Sample.ID
         +(1|Date.of.Infection)
         +(1|Date.of.Flow.Cytometry), data=cc1)
>   summary(m_g2)
Linear mixed model fit by REML t-tests use Satterthwaite approximations to
degrees of freedom [lmerMod]
Formula: G2Mlogit ~ Sample.ID + (1 | Date.of.Infection) + (1 | Date.of.Flo
w.Cytometry)
   Data: cc1

REML criterion at convergence: 6.8

Scaled residuals:
    Min      1Q  Median      3Q     Max
-1.5896 -0.4360 -0.0730  0.4398  1.5922

Random effects:
 Groups                 Name        Variance Std.Dev.
 Date.of.Infection      (Intercept) 0.002177 0.04666
 Date.of.Flow.Cytometry (Intercept) 0.008945 0.09458
 Residual                           0.072900 0.27000
Number of obs: 12, groups:  Date.of.Infection, 3; Date.of.Flow.Cytometry,
2

Fixed effects:
                 Estimate Std. Error      df t value Pr(>|t|)
(Intercept)       -2.3313     0.1727  4.1000 -13.502 0.000149 ***
Sample.IDNeg. C    0.2925     0.2205  6.0000   1.327 0.232783
Sample.IDPos. C.   1.4123     0.2205  6.0000   6.406 0.000682 ***
Sample.IDS5-0395   1.7650     0.2205  6.0000   8.006 0.000203 ***
---
Signif. codes:  0 ‘***’ 0.001 ‘**’ 0.01 ‘*’ 0.05 ‘.’ 0.1 ‘ ’ 1

Correlation of Fixed Effects:
            (Intr) S.IDNC S.IDPC
Smpl.IDNg.C -0.638
Smpl.IDP.C. -0.638  0.500
S.IDS5-0395 -0.638  0.500  0.500
>   anova(m_g2)
Analysis of Variance Table of type III  with  Satterthwaite
approximation for degrees of freedom
          Sum Sq Mean Sq NumDF DenDF F.value    Pr(>F)
Sample.ID 6.5565  2.1855     3     6  29.979 0.0005224 ***
---
Signif. codes:  0 ‘***’ 0.001 ‘**’ 0.01 ‘*’ 0.05 ‘.’ 0.1 ‘ ’ 1
>   ls.m_g2=lsmeans(m_g2,pairwise~Sample.ID)
>   cld(ls.m_g2)
 Sample.ID    lsmean        SE  df  lower.CL    upper.CL .group
 M8-0540   -2.3312757 0.1726613 4.1 -2.806093 -1.85645825  1
 Neg. C    -2.0387407 0.1726613 4.1 -2.513558 -1.56392321  1
```

```
 Pos. C.    -0.9189337 0.1726613 4.1 -1.393751 -0.44411618    2
 S5-0395    -0.5662829 0.1726613 4.1 -1.041100 -0.09146543    2

Degrees-of-freedom method: satterthwaite
Confidence level used: 0.95
P value adjustment: tukey method for comparing a family of 4 estimates
significance level used: alpha = 0.05
>   plot(resid(m_g2)~predict(m_g2))
```

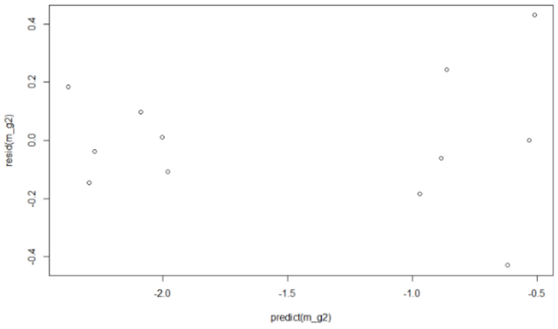


```
>   hist(resid(m_g2))
```

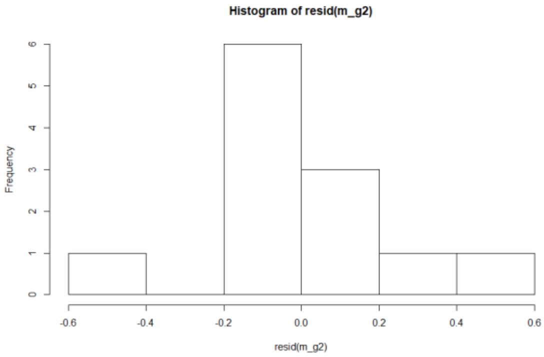


```
>   qqnorm(resid(m_g2))
>   qqline(resid(m_g2))
```

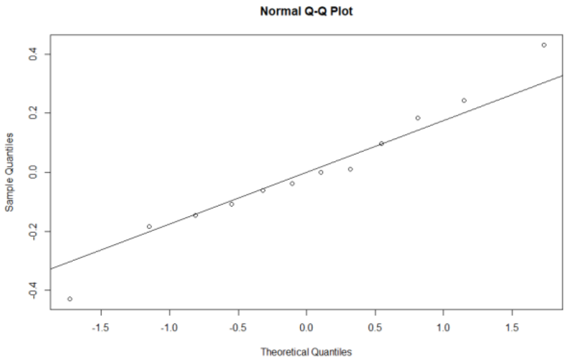


```
>   summary(ls.m_g2)
$lsmeans
 Sample.ID     lsmean        SE  df  lower.CL    upper.CL
 M8-0540    -2.3312757 0.1726613 4.1 -2.806093 -1.85645825
 Neg. C     -2.0387407 0.1726613 4.1 -2.513558 -1.56392321
 Pos. C.    -0.9189337 0.1726613 4.1 -1.393751 -0.44411618
 S5-0395    -0.5662829 0.1726613 4.1 -1.041100 -0.09146543

Degrees-of-freedom method: satterthwaite
Confidence level used: 0.95
```

```
$contrasts
 contrast             estimate        SE df t.ratio p.value
 M8-0540 - Neg. C   -0.2925350 0.2204547  6  -1.327  0.5806
 M8-0540 - Pos. C. -1.4123421 0.2204547  6  -6.406  0.0028
 M8-0540 - S5-0395 -1.7649928 0.2204547  6  -8.006  0.0008
 Neg. C - Pos. C.   -1.1198070 0.2204547  6  -5.080  0.0090
 Neg. C - S5-0395   -1.4724578 0.2204547  6  -6.679  0.0022
 Pos. C. - S5-0395 -0.3526508 0.2204547  6  -1.600  0.4442

P value adjustment: tukey method for comparing a family of 4 estimates

>   ###S
>   m_S=lmer((Slogit)~Sample.ID
+            +(1|Date.of.Infection)
+            +(1|Date.of.Flow.Cytometry), data=cc1)
>   summary(m_S)
Linear mixed model fit by REML t-tests use Satterthwaite approximations to
degrees of freedom [lmerMod]
Formula: (Slogit) ~ Sample.ID + (1 | Date.of.Infection) + (1 | Date.of.Flo
w.Cytometry)
   Data: cc1

REML criterion at convergence: 13.3

Scaled residuals:
     Min       1Q   Median       3Q      Max
-1.43123 -0.60060  0.07438  0.71562  1.00646

Random effects:
 Groups                 Name        Variance Std.Dev.
 Date.of.Infection      (Intercept) 0.0000   0.0000
 Date.of.Flow.Cytometry (Intercept) 0.0000   0.0000
 Residual                           0.1775   0.4213
Number of obs: 12, groups:  Date.of.Infection, 3; Date.of.Flow.Cytometry,
2

Fixed effects:
                 Estimate Std. Error      df t value Pr(>|t|)
(Intercept)       -1.3871     0.2432  8.0000  -5.703 0.000453 ***
Sample.IDNeg. C   -0.2124     0.3440  8.0000  -0.617 0.554094
Sample.IDPos. C.   0.3318     0.3440  8.0000   0.965 0.362971
Sample.IDS5-0395   0.5991     0.3440  8.0000   1.742 0.119744
Signif. codes:  0 ‘***’ 0.001 ‘**’ 0.01 ‘*’ 0.05 ‘.’ 0.1 ‘ ’ 1

Correlation of Fixed Effects:
            (Intr) S.IDNC S.IDPC
Smpl.IDNg.C -0.707
Smpl.IDP.C. -0.707  0.500
S.IDS5-0395 -0.707  0.500  0.500
>   anova(m_S)
Analysis of Variance Table of type III  with  Satterthwaite
approximation for degrees of freedom
          Sum Sq Mean Sq NumDF DenDF F.value Pr(>F)
Sample.ID 1.1552 0.38507     3     8  2.1696 0.1695
>   ls.m_S=lsmeans(m_S,pairwise~Sample.ID)
>   cld(ls.m_S)
 Sample.ID     lsmean        SE df  lower.CL   upper.CL .group
 Neg. C    -1.5994812 0.2432347  8 -2.160381 -1.0385810  1
 M8-0540   -1.3870826 0.2432347  8 -1.947983 -0.8261824  1
 Pos. C.   -1.0552572 0.2432347  8 -1.616157 -0.4943571  1
 S5-0395   -0.7879809 0.2432347  8 -1.348881 -0.2270808  1
```

```
Degrees-of-freedom method: satterthwaite
Results are given on the ( (not the response) scale.
Confidence level used: 0.95
P value adjustment: tukey method for comparing a family of 4 estimates
significance level used: alpha = 0.05
```

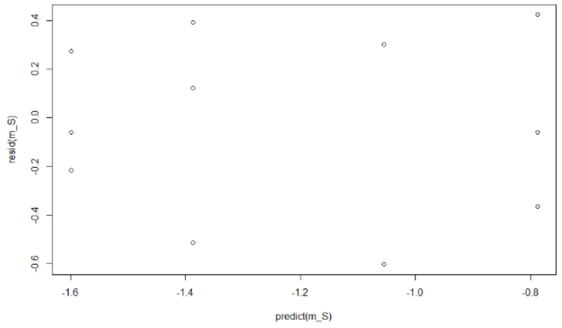


```
>   plot(resid(m_S)~predict(m_S))
>   hist(resid(m_S))
```

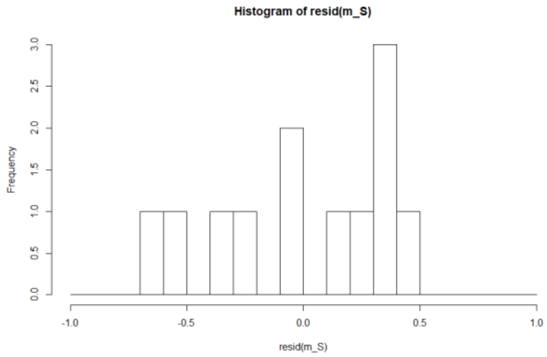


```
>   qqnorm(resid(m_S))
>   qqline(resid(m_S))
```

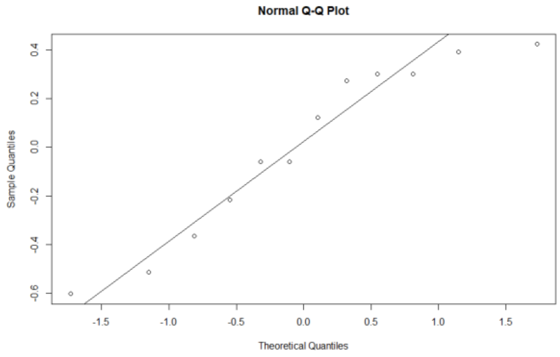


```
>   summary(ls.m_S)
$lsmeans
 Sample.ID     lsmean        SE df  lower.CL   upper.CL
 M8-0540   -1.3870826 0.2432347  8 -1.947983 -0.8261824
 Neg. C    -1.5994812 0.2432347  8 -2.160381 -1.0385810
 Pos. C.   -1.0552572 0.2432347  8 -1.616157 -0.4943571
 S5-0395   -0.7879809 0.2432347  8 -1.348881 -0.2270808

Degrees-of-freedom method: satterthwaite
Results are given on the ( (not the response) scale.
Confidence level used: 0.95

$contrasts
 contrast                estimate        SE df t.ratio p.value
```

```
 M8-0540 - Neg. C   0.2123986 0.3439858  8   0.617  0.9236
 M8-0540 - Pos. C. -0.3318253 0.3439858  8  -0.965  0.7725
 M8-0540 - S5-0395 -0.5991017 0.3439858  8  -1.742  0.3645
 Neg. C - Pos. C.  -0.5442239 0.3439858  8  -1.582  0.4387
 Neg. C - S5-0395  -0.8115002 0.3439858  8  -2.359  0.1633
 Pos. C. - S5-0395 -0.2672763 0.3439858  8  -0.777  0.8628

P value adjustment: tukey method for comparing a family of 4 estimates

>   ###G1
>   m_G1=lmer((G1logit)~Sample.ID
             +(1|Date.of.Infection)
             +(1|Date.of.Flow.Cytometry), data=cc1)
>   summary(m_G1)
Linear mixed model fit by REML t-tests use Satterthwaite approximations to
degrees of freedom [lmerMod]
Formula: (G1logit) ~ Sample.ID + (1 | Date.of.Infection) + (1 | Date.of.Fl
ow.Cytometry)
   Data: cc1

REML criterion at convergence: 2.3

Scaled residuals:
    Min      1Q  Median      3Q     Max
-1.4347 -0.4873  0.0041  0.5821  1.2829

Random effects:
 Groups                 Name        Variance  Std.Dev.
 Date.of.Infection      (Intercept) 4.952e-19 7.037e-10
 Date.of.Flow.Cytometry (Intercept) 1.416e-02 1.190e-01
 Residual                           3.944e-02 1.986e-01
Number of obs: 12, groups:  Date.of.Infection, 3; Date.of.Flow.Cytometry,
2

Fixed effects:
                 Estimate Std. Error       df t value Pr(>|t|)
(Intercept)       0.88534    0.14316  3.17500   6.184 0.007195 **
Sample.IDNeg. C   0.03869    0.16215  7.00000   0.239 0.818263
Sample.IDPos. C. -1.06416    0.16215  7.00000  -6.563 0.000315 ***
Sample.IDS5-0395 -1.63319    0.16215  7.00000 -10.072 2.04e-05 ***
---
Signif. codes:  0 '***' 0.001 '**' 0.01 '*' 0.05 '.' 0.1 ' ' 1

Correlation of Fixed Effects:
            (Intr) S.IDNC S.IDPC
Smpl.IDNg.C -0.566
Smpl.IDP.C. -0.566  0.500
S.IDS5-0395 -0.566  0.500  0.500
>   anova(m_G1)
Analysis of Variance Table of type III  with  Satterthwaite
approximation for degrees of freedom
          Sum Sq Mean Sq NumDF DenDF F.value    Pr(>F)
Sample.ID 6.1024  2.0341     3     7  51.576 3.861e-05 ***
---
Signif. codes:  0 '***' 0.001 '**' 0.01 '*' 0.05 '.' 0.1 ' ' 1
>   ls.m_G1=lsmeans(m_G1,pairwise~Sample.ID)
>   cld(ls.m_G1)
 Sample.ID     lsmean        SE   df   lower.CL   upper.CL .group
 S5-0395   -0.7478540 0.1431623 3.18 -1.1895647 -0.3061434  1
 Pos. C.   -0.1788165 0.1431623 3.18 -0.6205271  0.2628942   2
 M8-0540    0.8853393 0.1431623 3.18  0.4436287  1.3270499    3
 Neg. C     0.9240258 0.1431623 3.18  0.4823152  1.3657364    3
```

```
Degrees-of-freedom method: satterthwaite
Results are given on the ( (not the response) scale.
Confidence level used: 0.95
P value adjustment: tukey method for comparing a family of 4 estimates
significance level used: alpha = 0.05
>   plot(resid(m_G1)~predict(m_G1))
```

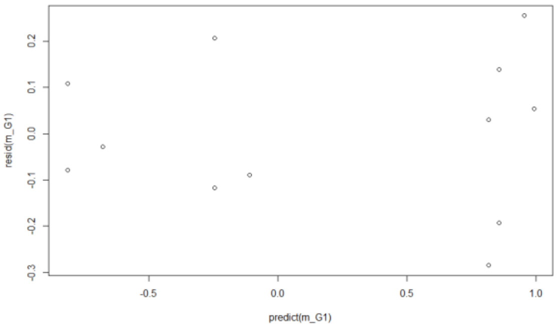

```
>   hist(resid(m_G1))
```

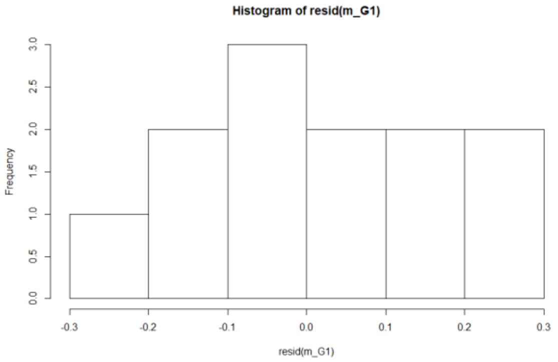


```
>   qqnorm(resid(m_G1))
>   qqline(resid(m_G1))
```

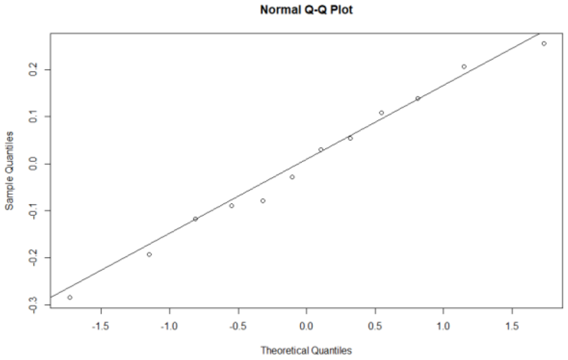


```
>   summary(ls.m_G1)
$lsmeans
 Sample.ID     lsmean        SE   df   lower.CL   upper.CL
 M8-0540    0.8853393 0.1431623 3.18  0.4436287  1.3270499
 Neg. C     0.9240258 0.1431623 3.18  0.4823152  1.3657364
 Pos. C.   -0.1788165 0.1431623 3.18 -0.6205271  0.2628942
 S5-0395   -0.7478540 0.1431623 3.18 -1.1895647 -0.3061434

Degrees-of-freedom method: satterthwaite
Results are given on the ( (not the response) scale.
Confidence level used: 0.95

$contrasts
 contrast                 estimate        SE df t.ratio p.value
 M8-0540 - Neg. C   -0.03868649 0.1621515  7  -0.239  0.9948
 M8-0540 - Pos. C.   1.06415576 0.1621515  7   6.563  0.0014
 M8-0540 - S5-0395   1.63319335 0.1621515  7  10.072  0.0001
 Neg. C - Pos. C.    1.10284225 0.1621515  7   6.801  0.0011
 Neg. C - S5-0395    1.67187984 0.1621515  7  10.311  0.0001
 Pos. C. - S5-0395   0.56903759 0.1621515  7   3.509  0.0387

P value adjustment: tukey method for comparing a family of 4 estimates

>
> ###LME MODEL FOR FIGURE 1D
>   if1=read.csv("IF_1.csv", sep=",", strip.white = TRUE, header=TRUE,na.s
trings="")
>   m_if1=lmer((logit)~FSL.ID+(1|Infection), data=if1)
>   anova(m_if1)
Analysis of Variance Table of type III  with  Satterthwaite
approximation for degrees of freedom
       Sum Sq Mean Sq NumDF DenDF F.value   Pr(>F)
FSL.ID 30.099  10.033     3     6  16.756 0.002544 **
```

```
---
Signif. codes:  0 '***' 0.001 '**' 0.01 '*' 0.05 '.' 0.1 ' ' 1
>   plot(predict(m_if1),resid(m_if1))
```

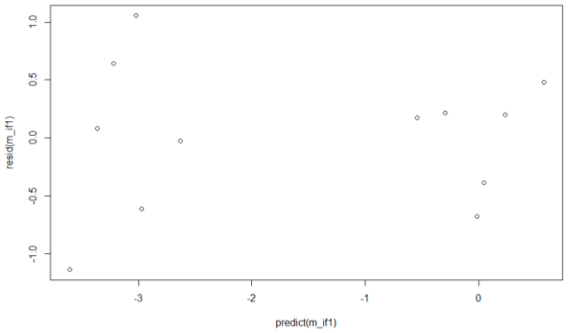


```
>   hist(resid(m_if1))
```

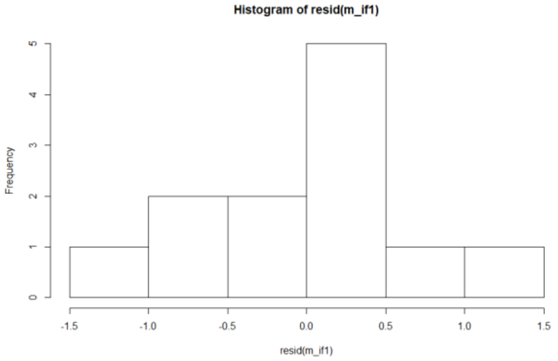


```
>   qqnorm(resid(m_if1))
>   qqline(resid(m_if1))
```

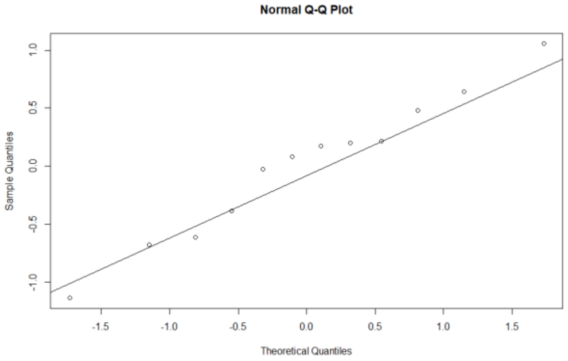


```
>   m_if1 <- lsmeans(m_if1,pairwise~FSL.ID)
>   cld(m_if1)
 FSL.ID               lsmean        SE   df   lower.CL   upper.CL .group
 Negative control -3.3285622 0.5048411 7.01 -4.5219564 -2.1351679  1
 M8-0540          -2.9387181 0.5048411 7.01 -4.1321124 -1.7453238  1
 S5-0395          -0.2651855 0.5048411 7.01 -1.4585798  0.9282087   2
 Positive control  0.2642889 0.5048411 7.01 -0.9291053  1.4576832   2

Degrees-of-freedom method: satterthwaite
Results are given on the ( (not the response) scale.
Confidence level used: 0.95
P value adjustment: tukey method for comparing a family of 4 estimates
significance level used: alpha = 0.05
```

```
>   summary(m_if1)
$lsmeans
 FSL.ID               lsmean        SE   df   lower.CL   upper.CL
 M8-0540          -2.9387181 0.5048411 7.01 -4.1321124 -1.7453238
 Negative control -3.3285622 0.5048411 7.01 -4.5219564 -2.1351679
 Positive control  0.2642889 0.5048411 7.01 -0.9291053  1.4576832
 S5-0395          -0.2651855 0.5048411 7.01 -1.4585798  0.9282087

Degrees-of-freedom method: satterthwaite
Results are given on the ( (not the response) scale.
Confidence level used: 0.95

$contrasts
 contrast                             estimate        SE df t.ratio p.value
 M8-0540 - Negative control          0.3898441 0.6317998  6   0.617  0.9230
 M8-0540 - Positive control         -3.2030070 0.6317998  6  -5.070  0.0091
 M8-0540 - S5-0395                  -2.6735325 0.6317998  6  -4.232  0.0213
 Negative control-Positive control -3.5928511 0.6317998  6  -5.687  0.0051
 Negative control-S5-0395          -3.0633766 0.6317998  6  -4.849  0.0113
 Positive control-S5-0395           0.5294745 0.6317998  6   0.838  0.8351

P value adjustment: tukey method for comparing a family of 4 estimates

>
> ###IF MODEL FOR FIGURE 2C
>   if2=read.csv("F2_IF_7NOV17.csv", sep=",", strip.white = TRUE, header=T
RUE,na.strings="")
>   m_if2=lmer((logit)~FSL.ID+(1|Infection), data=if2)
>   anova(m_if2)
Analysis of Variance Table of type III  with  Satterthwaite
approximation for degrees of freedom
       Sum Sq Mean Sq NumDF  DenDF F.value    Pr(>F)
FSL.ID 42.795  7.1325     6 13.578  12.006 9.462e-05 ***
---
Signif. codes:  0 ‘***’ 0.001 ‘**’ 0.01 ‘*’ 0.05 ‘.’ 0.1 ‘ ’ 1
>   plot(predict(m_if2),resid(m_if2))
```

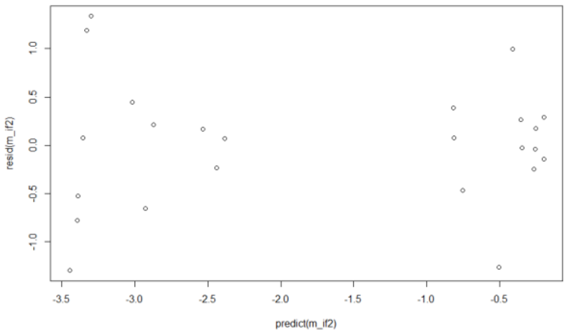


```
>   hist(resid(m_if2))
```

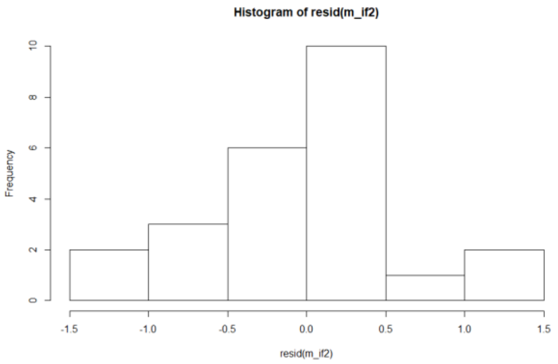


```
>   qqnorm(resid(m_if2))
>   qqline(resid(m_if2))
```

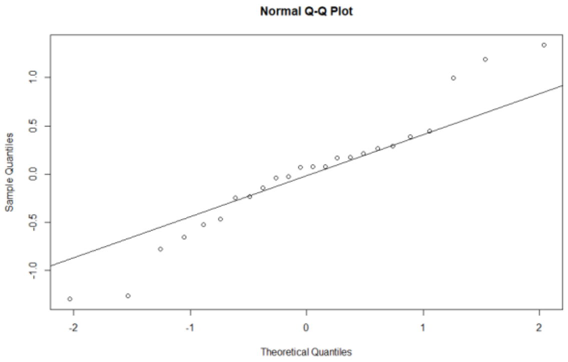


```
>   m_if2 <- lsmeans(m_if2,pairwise~FSL.ID)
>   cld(m_if2)
 FSL.ID                lsmean        SE    df  lower.CL   upper.CL .group
 Negative control -3.3686938 0.3208995 16.94 -4.045803 -2.6915844  1
 M8-0540          -2.9402638 0.4536521 16.98 -3.897486 -1.9830416  1
 M8-0532          -2.4558643 0.4536521 16.98 -3.413087 -1.4986422  12
 M8-0577          -0.7921536 0.4536521 16.98 -1.749376  0.1650686   23
 M8-0533          -0.4256290 0.4536521 16.98 -1.382851  0.5315932   23
 S5-0395          -0.2667312 0.4536521 16.98 -1.223953  0.6904910    3
 M8-0578          -0.2383565 0.4536521 16.98 -1.195579  0.7188657    3

Degrees-of-freedom method: satterthwaite
Results are given on the ( (not the response) scale.
Confidence level used: 0.95
P value adjustment: tukey method for comparing a family of 7 estimates
significance level used: alpha = 0.05
>   summary(m_if2)
$lsmeans
 FSL.ID                lsmean        SE    df  lower.CL   upper.CL
 M8-0532          -2.4558643 0.4536521 16.98 -3.413087 -1.4986422
 M8-0533          -0.4256290 0.4536521 16.98 -1.382851  0.5315932
 M8-0540          -2.9402638 0.4536521 16.98 -3.897486 -1.9830416
 M8-0577          -0.7921536 0.4536521 16.98 -1.749376  0.1650686
 M8-0578          -0.2383565 0.4536521 16.98 -1.195579  0.7188657
 Negative control -3.3686938 0.3208995 16.94 -4.045803 -2.6915844
 S5-0395          -0.2667312 0.4536521 16.98 -1.223953  0.6904910

Degrees-of-freedom method: satterthwaite
Results are given on the ( (not the response) scale.
Confidence level used: 0.95
```

```
$contrasts
 contrast                        estimate        SE    df t.ratio p.value
 M8-0532 - M8-0533            -2.03023538 0.6293181 13.63  -3.226  0.0711
 M8-0532 - M8-0540             0.48439942 0.6293181 13.63   0.770  0.9843
 M8-0532 - M8-0577            -1.66371079 0.6413228 17.00  -2.594  0.1882
 M8-0532 - M8-0578            -2.21750788 0.6413228 17.00  -3.458  0.0388
 M8-0532 - Negative control    0.91282949 0.5484928 15.40   1.664  0.6469
 M8-0532 - S5-0395            -2.18913312 0.6293181 13.63  -3.479  0.0458
 M8-0533 - M8-0540             2.51463481 0.6293181 13.63   3.996  0.0182
 M8-0533 - M8-0577             0.36652460 0.6413228 17.00   0.572  0.9969
 M8-0533 - M8-0578            -0.18727249 0.6413228 17.00  -0.292  0.9999
 M8-0533 - Negative control    2.94306487 0.5484928 15.40   5.366  0.0011
 M8-0533 - S5-0395            -0.15889774 0.6293181 13.63  -0.252  1.0000
 M8-0540 - M8-0577            -2.14811021 0.6413228 17.00  -3.349  0.0479
 M8-0540 - M8-0578            -2.70190730 0.6413228 17.00  -4.213  0.0085
 M8-0540 - Negative control    0.42843007 0.5484928 15.40   0.781  0.9835
 M8-0540 - S5-0395            -2.67353255 0.6293181 13.63  -4.248  0.0116
 M8-0577 - M8-0578            -0.55379709 0.6293181 13.63  -0.880  0.9698
 M8-0577 - Negative control    2.57654028 0.5484928 15.40   4.697  0.0040
 M8-0577 - S5-0395            -0.52542234 0.6413228 17.00  -0.819  0.9795
 M8-0578 - Negative control    3.13033737 0.5484928 15.40   5.707  0.0006
 M8-0578 - S5-0395             0.02837475 0.6413228 17.00   0.044  1.0000
 Negative control - S5-0395   -3.10196261 0.5484928 15.40  -5.655  0.0007

P value adjustment: tukey method for comparing a family of 7 estimates

>
> ###IF MODEL FOR FIGURE 3C
>   if3=read.csv("IF_3.1.csv", sep=",", strip.white = TRUE, header=TRUE,na
.strings="")
>   m_if3=lmer((logit)~FSL.ID+(1|Infection)+ 1:FSL.ID:Infection, data=if3)
>   anova(m_if3)
Analysis of Variance Table of type III  with  Satterthwaite
approximation for degrees of freedom
       Sum Sq Mean Sq NumDF  DenDF F.value    Pr(>F)
FSL.ID 33.694  5.6157     6 13.476   10.54 0.0001976 ***
---
Signif. codes:  0 ‘***’ 0.001 ‘**’ 0.01 ‘*’ 0.05 ‘.’ 0.1 ‘ ’ 1
>   plot(predict(m_if3),resid(m_if3))
```

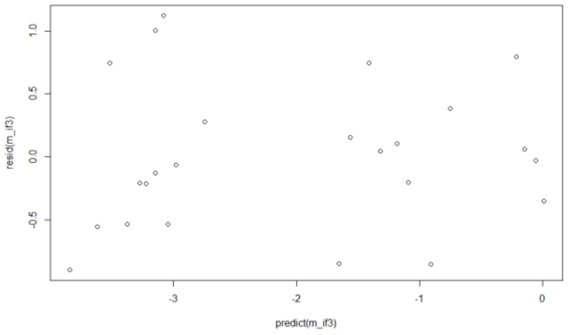


```
>   hist(resid(m_if3))
```

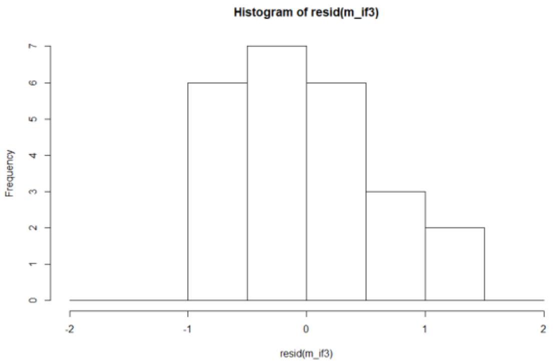


```
>   qqnorm(resid(m_if3))
>   qqline(resid(m_if3))
```

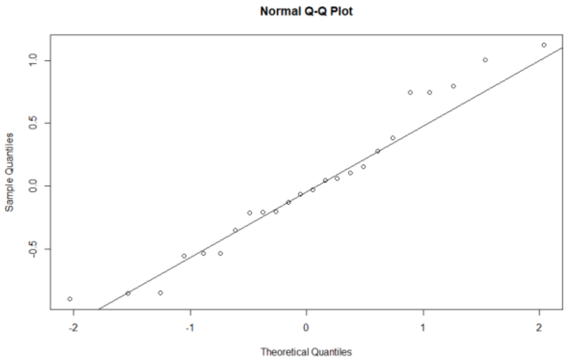


```
>   m_if3 <- lsmeans(m_if3,pairwise~FSL.ID)
>   cld(m_if3)
 FSL.ID               lsmean        SE    df  lower.CL   upper.CL .group
 Negative control -3.3686938 0.3457506 14.14 -4.101305 -2.6360829  1
 M8-0585          -3.2687963 0.4808144 16.09 -4.287594 -2.2499989  12
 M8-0586          -2.9718834 0.4808144 16.09 -3.990681 -1.9530861  12
 M8-0582          -1.4066186 0.4808144 16.09 -2.425416 -0.3878212   23
 M8-0583          -1.3138963 0.4808144 16.09 -2.332694 -0.2950989   23
 M8-0533          -0.4344029 0.4808144 16.09 -1.453200  0.5843945    3
 S5-0395          -0.2755052 0.4808144 16.09 -1.294303  0.7432922    3

Results are averaged over the levels of: Infection
Degrees-of-freedom method: satterthwaite
Results are given on the ( (not the response) scale.
Confidence level used: 0.95
P value adjustment: tukey method for comparing a family of 7 estimates
significance level used: alpha = 0.05
>   summary(m_if3)
$lsmeans
 FSL.ID               lsmean        SE    df  lower.CL   upper.CL
 M8-0533          -0.4344029 0.4808144 16.09 -1.453200  0.5843945
 M8-0582          -1.4066186 0.4808144 16.09 -2.425416 -0.3878212
 M8-0583          -1.3138963 0.4808144 16.09 -2.332694 -0.2950989
 M8-0585          -3.2687963 0.4808144 16.09 -4.287594 -2.2499989
 M8-0586          -2.9718834 0.4808144 16.09 -3.990681 -1.9530861
 Negative control -3.3686938 0.3457506 14.14 -4.101305 -2.6360829
 S5-0395          -0.2755052 0.4808144 16.09 -1.294303  0.7432922

Results are averaged over the levels of: Infection
Degrees-of-freedom method: satterthwaite
Results are given on the ( (not the response) scale.
Confidence level used: 0.95

$contrasts
```

```
 contrast                     estimate        SE    df t.ratio p.value
 M8-0533 - M8-0582          0.97221567 0.6682482 17.00   1.455  0.7659
 M8-0533 - M8-0583          0.87949337 0.6682482 17.00   1.316  0.8355
 M8-0533 - M8-0585          2.83439341 0.6682482 17.00   4.242  0.0080
 M8-0533 - M8-0586          2.53748055 0.6682482 17.00   3.797  0.0198
 M8-0533 - Negative control  2.93429094 0.5378161 14.85   5.456  0.0011
 M8-0533 - S5-0395         -0.15889774 0.5959987 12.21  -0.267  1.0000
 M8-0582 - M8-0583         -0.09272230 0.5959987 12.21  -0.156  1.0000
 M8-0582 - M8-0585          1.86217774 0.5959987 12.21   3.124  0.0912
 M8-0582 - M8-0586          1.56526488 0.5959987 12.21   2.626  0.1988
 M8-0582 - Negative control  1.96207527 0.5378161 14.85   3.648  0.0308
 M8-0582 - S5-0395         -1.13111341 0.6682482 17.00  -1.693  0.6294
 M8-0583 - M8-0585          1.95490004 0.5959987 12.21   3.280  0.0707
 M8-0583 - M8-0586          1.65798718 0.5959987 12.21   2.782  0.1571
 M8-0583 - Negative control  2.05479757 0.5378161 14.85   3.821  0.0223
 M8-0583 - S5-0395         -1.03839111 0.6682482 17.00  -1.554  0.7109
 M8-0585 - M8-0586         -0.29691286 0.5959987 12.21  -0.498  0.9984
 M8-0585 - Negative control  0.09989753 0.5378161 14.85   0.186  1.0000
 M8-0585 - S5-0395         -2.99329115 0.6682482 17.00  -4.479  0.0050
 M8-0586 - Negative control  0.39681039 0.5378161 14.85   0.738  0.9876
 M8-0586 - S5-0395         -2.69637829 0.6682482 17.00  -4.035  0.0122
 Negative control - S5-0395 -3.09318868 0.5378161 14.85  -5.751  0.0006

Results are averaged over the levels of: Infection
P value adjustment: tukey method for comparing a family of 7 estimates

> ###LMER MODEL FOR FIGURE 5: GAMMA-H2AX POSITIVE CELLS BY CELL CYCLE PHAS
E
>   H2AXpos= read.csv("cyclegammaH2AX.csv", sep=",",strip.white = TRUE, he
ader=TRUE)
>   m_h2ax=lmer(logit~(1|Date)+
          Treatment+
          Phase+
          (1|Date:Treatment)+
          (1|Date:Phase)+
          Treatment:Phase,
        data=H2AXpos)
>   anova(m_h2ax)
Analysis of Variance Table of type III  with  Satterthwaite
approximation for degrees of freedom
                Sum Sq Mean Sq NumDF  DenDF F.value    Pr(>F)    
Treatment        0.500  0.1668     3 4.0002   22.34  0.005832 ** 
Phase           37.282 18.6411     2 8.0002 2497.15 6.538e-12 ***
Treatment:Phase  6.007  1.0012     6 8.0002  134.12 1.386e-07 ***
---
Signif. codes:  0 ‘***’ 0.001 ‘**’ 0.01 ‘*’ 0.05 ‘.’ 0.1 ‘ ’ 1
>   plot(predict(m_h2ax),resid(m_h2ax))
```

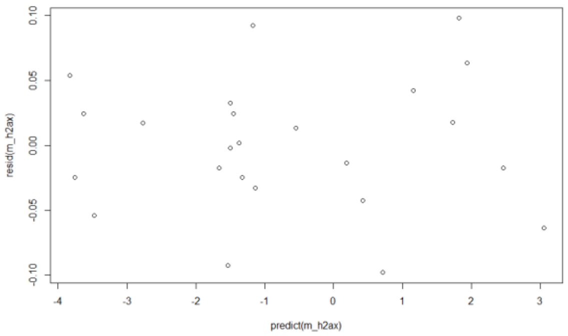


```
>   hist(resid(m_h2ax))
```

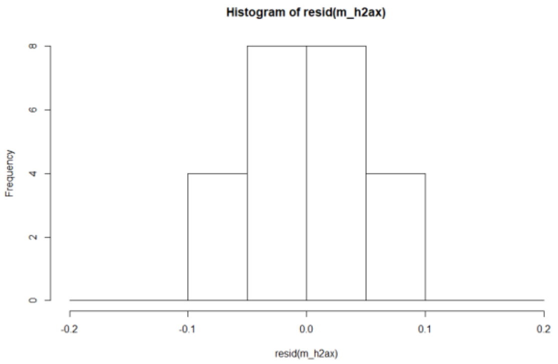


```
>   qqnorm(resid(m_h2ax))
>   qqline(resid(m_h2ax))
```

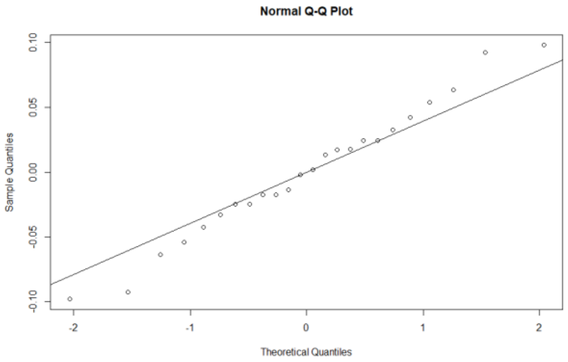


```
>   m_lsm <- lsmeans(m_h2ax,pairwise~Treatment|Phase)
>   summary(m_lsm)
$lsmeans
Phase = G1:
 Treatment     lsmean        SE   df   lower.CL   upper.CL
 395        -2.2179231 0.3535286 4.16 -3.1844344 -1.2514117
 540        -3.6554000 0.3535286 4.16 -4.6219113 -2.6888886
 Neg.       -3.6989177 0.3535286 4.16 -4.6654291 -2.7324064
 Pos.       -0.1765573 0.3535286 4.16 -1.1430686  0.7899541

Phase = G2/M:
 Treatment     lsmean        SE   df   lower.CL   upper.CL
 395         2.4967456 0.3535286 4.16  1.5302343  3.4632570
 540        -1.3156028 0.3535286 4.16 -2.2821141 -0.3490914
 Neg.       -1.3889793 0.3535286 4.16 -2.3554907 -0.4224680
 Pos.        2.0983125 0.3535286 4.16  1.1318011  3.0648238

Phase = S:
 Treatment     lsmean        SE   df   lower.CL   upper.CL
 395         1.2670326 0.3535286 4.16  0.3005212  2.2335439
 540        -1.3567666 0.3535286 4.16 -2.3232779 -0.3902552
 Neg.       -1.4332876 0.3535286 4.16 -2.3997989 -0.4667762
 Pos.        0.7941917 0.3535286 4.16 -0.1723196  1.7607031

Degrees-of-freedom method: satterthwaite
Confidence level used: 0.95

$contrasts
Phase = G1:
 contrast       estimate        SE   df t.ratio p.value
 395 - 540    1.43747692 0.4999649 4.16   2.875  0.1350
 395 - Neg.   1.48099467 0.4999649 4.16   2.962  0.1243
 395 - Pos.  -2.04136582 0.4999649 4.16  -4.083  0.0464
 540 - Neg.   0.04351776 0.4999649 4.16   0.087  0.9997
```

```
 540 - Pos.  -3.47884273 0.4999649 4.16  -6.958  0.0068
 Neg. - Pos. -3.52236049 0.4999649 4.16  -7.045  0.0065

Phase = G2/M:
 contrast       estimate        SE   df t.ratio p.value
 395 - 540    3.81234842 0.4999649 4.16   7.625  0.0048
 395 - Neg.   3.88572497 0.4999649 4.16   7.772  0.0044
 395 - Pos.   0.39843316 0.4999649 4.16   0.797  0.8532
 540 - Neg.   0.07337655 0.4999649 4.16   0.147  0.9987
 540 - Pos.  -3.41391526 0.4999649 4.16  -6.828  0.0073
 Neg. - Pos. -3.48729181 0.4999649 4.16  -6.975  0.0067

Phase = S:
 contrast       estimate        SE   df t.ratio p.value
 395 - 540    2.62379916 0.4999649 4.16   5.248  0.0194
 395 - Neg.   2.70032014 0.4999649 4.16   5.401  0.0175
 395 - Pos.   0.47284086 0.4999649 4.16   0.946  0.7848
 540 - Neg.   0.07652098 0.4999649 4.16   0.153  0.9985
 540 - Pos.  -2.15095830 0.4999649 4.16  -4.302  0.0389
 Neg. - Pos. -2.22747928 0.4999649 4.16  -4.455  0.0346

P value adjustment: tukey method for comparing a family of 4 estimates

>   cld(m_lsm)
Phase = G1:
 Treatment     lsmean        SE   df   lower.CL   upper.CL .group
 Neg.      -3.6989177 0.3535286 4.16 -4.6654291 -2.7324064  1
 540       -3.6554000 0.3535286 4.16 -4.6219113 -2.6888886  1
 395       -2.2179231 0.3535286 4.16 -3.1844344 -1.2514117  1
 Pos.      -0.1765573 0.3535286 4.16 -1.1430686  0.7899541   2

Phase = G2/M:
 Treatment     lsmean        SE   df   lower.CL   upper.CL .group
 Neg.      -1.3889793 0.3535286 4.16 -2.3554907 -0.4224680  1
 540       -1.3156028 0.3535286 4.16 -2.2821141 -0.3490914  1
 Pos.       2.0983125 0.3535286 4.16  1.1318011  3.0648238   2
 395        2.4967456 0.3535286 4.16  1.5302343  3.4632570   2

Phase = S:
 Treatment     lsmean        SE   df   lower.CL   upper.CL .group
 Neg.      -1.4332876 0.3535286 4.16 -2.3997989 -0.4667762  1
 540       -1.3567666 0.3535286 4.16 -2.3232779 -0.3902552  1
 Pos.       0.7941917 0.3535286 4.16 -0.1723196  1.7607031   2
 395        1.2670326 0.3535286 4.16  0.3005212  2.2335439   2

Degrees-of-freedom method: satterthwaite
Confidence level used: 0.95
P value adjustment: tukey method for comparing a family of 4 estimates
significance level used: alpha = 0.05
>
> ###LMER MODEL FOR FIGURE 6B-6C: ANNEXIN V AND PI POSITIVE CELLS
> PI488= read.csv("Annexin_PI_488.csv", sep=",",strip.white = TRUE, header
=TRUE)
>   ###Annexinv pos model
>   A488_pos<-lmer(Annexin_logit~(1|Date.of.Flow.Cytometry)
+                  +Treatment
+                  +(1:Date.of.Flow.Cytometry:Treatment), data=PI488)
>   anova(A488_pos)
Analysis of Variance Table of type III  with  Satterthwaite
approximation for degrees of freedom
          Sum Sq Mean Sq NumDF DenDF F.value    Pr(>F)
Treatment  21.14  7.0465     3    17  17.265 2.093e-05 ***
```

```
---
Signif. codes:  0 ‘***’ 0.001 ‘**’ 0.01 ‘*’ 0.05 ‘.’ 0.1 ‘ ’ 1
>   plot(predict(A488_pos),resid(A488_pos))
```

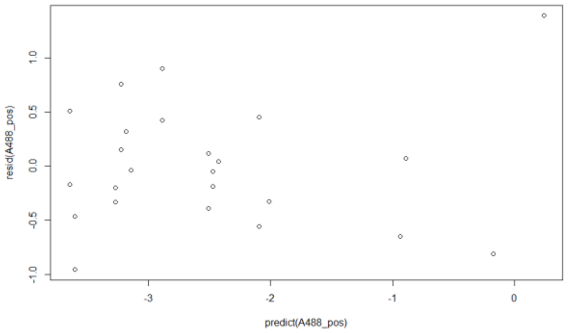

```
>   hist(resid(A488_pos))
```

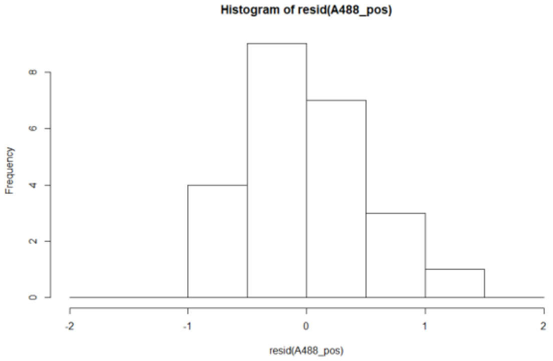

```
>   qqnorm(resid(A488_pos))
>   qqline(resid(A488_pos))
```

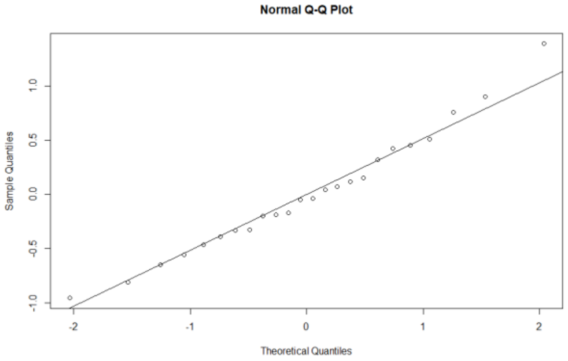

```
>   A488_lsm <- lsmeans(A488_pos,pairwise~Treatment)
>   cld(A488_lsm)
 Treatment         lsmean        SE   df  lower.CL  upper.CL .group
 M8-0540      -3.1513839 0.3835357 5.00 -4.029025 -2.273742  1
 S5-0395      -2.7743824 0.3835357 5.00 -3.652024 -1.896741  1
 Neg. Control -2.6905262 0.4451027 8.37 -3.709051 -1.672001  1
 5 uM H2O2    -0.4385738 0.4451027 8.37 -1.457099  0.579951   2

Results are averaged over the levels of: Date.of.Flow.Cytometry
Degrees-of-freedom method: satterthwaite
Confidence level used: 0.95
P value adjustment: tukey method for comparing a family of 4 estimates
significance level used: alpha = 0.05
>   summary(A488_lsm)
$lsmeans
 Treatment         lsmean        SE   df  lower.CL  upper.CL
 5 uM H2O2    -0.4385738 0.4451027 8.37 -1.457099  0.579951
```

```
 M8-0540       -3.1513839 0.3835357 5.00 -4.029025 -2.273742
 Neg. Control -2.6905262 0.4451027 8.37 -3.709051 -1.672001
 S5-0395      -2.7743824 0.3835357 5.00 -3.652024 -1.896741

Results are averaged over the levels of: Date.of.Flow.Cytometry
Degrees-of-freedom method: satterthwaite
Confidence level used: 0.95

$contrasts
 contrast                     estimate        SE df t.ratio p.value
 5 uM H2O2 - M8-0540        2.71281009 0.3912163 17   6.934  <.0001
 5 uM H2O2 - Neg. Control   2.25195243 0.4517377 17   4.985  0.0006
 5 uM H2O2 - S5-0395        2.33580859 0.3912163 17   5.971  0.0001
 M8-0540 - Neg. Control    -0.46085766 0.3912163 17  -1.178  0.6482
 M8-0540 - S5-0395         -0.37700150 0.3194268 17  -1.180  0.6469
 Neg. Control - S5-0395     0.08385616 0.3912163 17   0.214  0.9964

Results are averaged over the levels of: Date.of.Flow.Cytometry
P value adjustment: tukey method for comparing a family of 4 estimates

>
>   ###PI positive model
>   PI_pos<-lmer(PI_logit~(1|Date.of.Flow.Cytometry)
+                +Treatment
+                +(1:Date.of.Flow.Cytometry:Treatment), data=PI488)
>   anova(PI_pos)
Analysis of Variance Table of type III  with  Satterthwaite
approximation for degrees of freedom
          Sum Sq Mean Sq NumDF DenDF F.value    Pr(>F)
Treatment 46.519  15.506     3    20  27.482 2.699e-07 ***
---
Signif. codes:  0 ‘***’ 0.001 ‘**’ 0.01 ‘*’ 0.05 ‘.’ 0.1 ‘ ’ 1
>   plot(predict(PI_pos),resid(PI_pos))
```

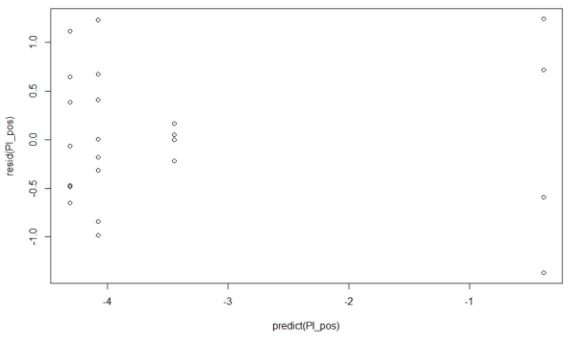


```
>   hist(resid(PI_pos))
```

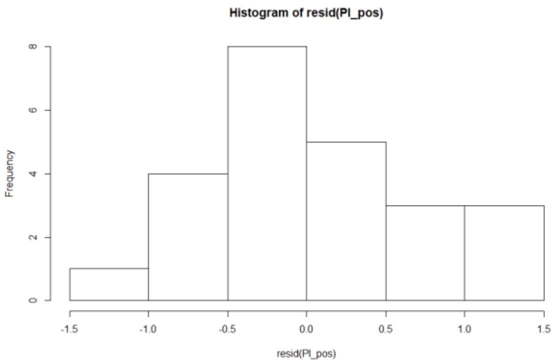


```
>   qqnorm(resid(PI_pos))
>   qqline(resid(PI_pos))
```

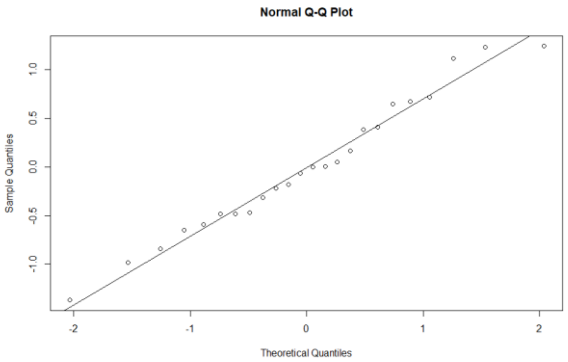


```
>   PI_lsm <- lsmeans(PI_pos,pairwise~Treatment)
>   cld(PI_lsm)
 Treatment        lsmean        SE df  lower.CL   upper.CL .group
 M8-0540      -4.310037 0.2655741 20 -4.864015 -3.7560591  1
 S5-0395      -4.073794 0.2655741 20 -4.627772 -3.5198162  1
 Neg. Control -3.447397 0.3755785 20 -4.230841 -2.6639543  1
 5 uM H2O2    -0.388357 0.3755785 20 -1.171800  0.3950861   2

Results are averaged over the levels of: Date.of.Flow.Cytometry
Degrees-of-freedom method: satterthwaite
Confidence level used: 0.95
P value adjustment: tukey method for comparing a family of 4 estimates
significance level used: alpha = 0.05
>   summary(PI_lsm)
$lsmeans
 Treatment        lsmean        SE df  lower.CL   upper.CL
 5 uM H2O2    -0.388357 0.3755785 20 -1.171800  0.3950861
 M8-0540      -4.310037 0.2655741 20 -4.864015 -3.7560591
 Neg. Control -3.447397 0.3755785 20 -4.230841 -2.6639543
 S5-0395      -4.073794 0.2655741 20 -4.627772 -3.5198162

Results are averaged over the levels of: Date.of.Flow.Cytometry
Degrees-of-freedom method: satterthwaite
Confidence level used: 0.95

$contrasts
 contrast                    estimate        SE df t.ratio p.value
 5 uM H2O2 - M8-0540       3.9216800 0.4599879 20   8.526  <.0001
 5 uM H2O2 - Neg. Control  3.0590404 0.5311483 20   5.759  0.0001
 5 uM H2O2 - S5-0395       3.6854371 0.4599879 20   8.012  <.0001
 M8-0540 - Neg. Control   -0.8626396 0.4599879 20  -1.875  0.2697
 M8-0540 - S5-0395        -0.2362429 0.3755785 20  -0.629  0.9215
 Neg. Control - S5-0395    0.6263967 0.4599879 20   1.362  0.5365

Results are averaged over the levels of: Date.of.Flow.Cytometry
P value adjustment: tukey method for comparing a family of 4 estimates

> ###Pathology scores for Figure 7
>   path=read.csv("pathologyscores.csv", sep=",", strip.white=TRUE,header=
TRUE)
>   kruskal.test(Ce_PMN~Strain,data=path)

        Kruskal-Wallis rank sum test

data:  Ce_PMN by Strain
Kruskal-Wallis chi-squared = 3.888, df = 1, p-value = 0.04863

>   ###p-value = 0.04863
>
```

```
>   kruskal.test(Cryptitis~Strain,data=path)

        Kruskal-Wallis rank sum test

data:  Cryptitis by Strain
Kruskal-Wallis chi-squared = 2.4143, df = 1, p-value = 0.1202

>   ###p-value = 0.1202
>
>   kruskal.test(Edema~Strain,data=path)

        Kruskal-Wallis rank sum test

data:  Edema by Strain
Kruskal-Wallis chi-squared = 1.452, df = 1, p-value = 0.2282

>   ###p-value = 0.2282
>
>   kruskal.test(Co_PMN~Strain, data=path)

        Kruskal-Wallis rank sum test

data:  Co_PMN by Strain
Kruskal-Wallis chi-squared = 1.1571, df = 1, p-value = 0.2821

>   ###p-value = 0.2821
>
>   pathtotal=read.csv("Path.csv", sep=",", strip.white=TRUE,header=TRUE)
>   kruskal.test(Total~Group,data=pathtotal)###p-value = 0.07491

        Kruskal-Wallis rank sum test

data:  Total by Group
Kruskal-Wallis chi-squared = 3.172, df = 1, p-value = 0.07491


>
>   ###Supplementary data: Rationale for not adjusting for organ weight
>     library(outliers)
>     ###Outliers in Spleen Weights
>     boxplot(c(0.079,0.141,0.092,0.097,0.089,0.086,0.101,0.083,0.103,0.08
7))
```

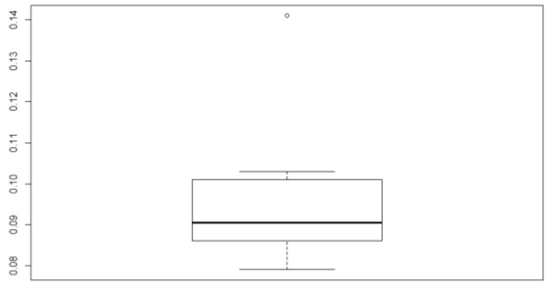


```
>     grubbs.test(c(0.079,0.141,0.092,0.097,0.089,0.086,0.101,0.083,0.103,
0.087))

        Grubbs test for one outlier

data:  c(0.079, 0.141, 0.092, 0.097, 0.089, 0.086, 0.101, 0.083, 0.103,
0.087)
```

```
G = 2.56100, U = 0.19031, p-value = 0.001949
alternative hypothesis: highest value 0.141 is an outlier

>     #data:  c(0.079, 0.141, 0.092, 0.097, 0.089, 0.086, 0.101, 0.083, 0.
103,0.087)
>     #G = 2.56100, U = 0.19031, p-value = 0.001949
>     #alternative hypothesis: highest value 0.141 is an outlier
>
>     ###Outliers in Liver Weights
>     grubbs.test(c(0.911,1.141,0.878,1.056,0.874,0.999,0.812,1.078,0.922,
1.074))

        Grubbs test for one outlier

data:  c(0.911, 1.141, 0.878, 1.056, 0.874, 0.999, 0.812, 1.078, 0.922,
1.074)
G = 1.51860, U = 0.71528, p-value = 0.5609
alternative hypothesis: highest value 1.141 is an outlier

>  #data:  c(0.911, 1.141, 0.878, 1.056, 0.874, 0.999, 0.812, 1.078, 0.922
,1.074)
>     #G = 1.51860, U = 0.71528, p-value = 0.5609
>     #alternative hypothesis: highest value 1.141 is an outlier
>   boxplot(c(0.911,1.141,0.878,1.056,0.874,0.999,0.812,1.078,0.922,1.074)
```

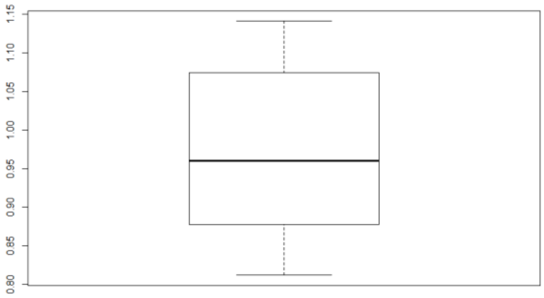


```
>     ### Outliers in FC in Ileum weights
>     ###boxplot FC Ileum
>     boxplot(c(0.135,0.056,0.062,0.118,0.064,0.127,0.125,0.104,0.07,0.102
))

>     grubbs.test(c(0.135,0.056,0.062,0.118,0.064,0.127,0.125,0.104,0.07,0
.102))

        Grubbs test for one outlier

data:  c(0.135, 0.056, 0.062, 0.118, 0.064, 0.127, 0.125, 0.104, 0.07,
0.102)
G = 1.3221, U = 0.7842, p-value = 0.8809
alternative hypothesis: lowest value 0.056 is an outlier

>     #data:  c(0.135, 0.056, 0.062, 0.118, 0.064, 0.127, 0.125, 0.104, 0.
07,0.102)
>     #G = 1.3221, U = 0.7842, p-value = 0.8809
>     #alternative hypothesis: lowest value 0.056 is an outlier

>     ###Outliers in FC in Cecum weights
>     ###boxplot FC cecum
```

```
>     boxplot(c(0.127,0.171,0.123,0.134,0.151,0.166,0.121,0.198,0.168,0.14
```

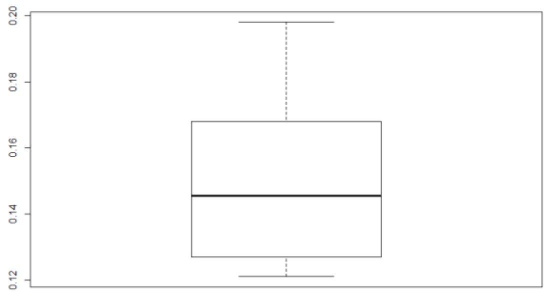


```
))

>     grubbs.test(c(0.127,0.171,0.123,0.134,0.151,0.166,0.121,0.198,0.168,
0.14))

        Grubbs test for one outlier

data:  c(0.127, 0.171, 0.123, 0.134, 0.151, 0.166, 0.121, 0.198, 0.168,
0.14)
G = 1.89790, U = 0.55532, p-value = 0.176
alternative hypothesis: highest value 0.198 is an outlier

>     #data:  c(0.127, 0.171, 0.123, 0.134, 0.151, 0.166, 0.121, 0.198, 0.
168,0.14)
>     #G = 1.89790, U = 0.55532, p-value = 0.176
>     #alternative hypothesis: highest value 0.198 is an outlier
>
>   ### Supplementary data: Invasion differences in WT and delta cdtB
>   data3=read.csv("WT_deltacdtB.csv",sep=",",strip.white=TRUE,header=TRUE
)
>   lme3=lmer(logit ~Strain +(1|Infection), data=data3)###significant effe
ct of Invasion.Assay (necessary to control)
>   summary(lme3)
Linear mixed model fit by REML t-tests use Satterthwaite approximations to
degrees of freedom [
lmerMod]
Formula: logit ~ Strain + (1 | Infection)
   Data: data3

REML criterion at convergence: 23

Scaled residuals:
     Min       1Q   Median       3Q      Max
-1.21699 -0.51998 -0.06481  0.54830  1.51408

Random effects:
 Groups    Name        Variance Std.Dev.
 Infection (Intercept) 0.6138   0.7834
 Residual              0.3292   0.5737
Number of obs: 11, groups:  Infection, 3

Fixed effects:
              Estimate Std. Error      df t value Pr(>|t|)
(Intercept)     0.6558     0.5094  2.3640   1.287    0.310
StrainS5-0395  -0.1352     0.3508  6.9920  -0.385    0.711

Correlation of Fixed Effects:
            (Intr)
StrnS5-0395 -0.307
```

```
>   anova(lme3)
Analysis of Variance Table of type III  with  Satterthwaite
approximation for degrees of freedom
         Sum Sq  Mean Sq NumDF  DenDF F.value Pr(>F)
Strain 0.048915 0.048915     1 6.9925 0.14861 0.7113
>   lsmeans(lme3,pairwise~Strain)
$lsmeans
 Strain     lsmean        SE   df  lower.CL upper.CL
 M8-0540 0.6558046 0.5093658 2.36 -1.242400 2.554009
 S5-0395 0.5205833 0.5222783 2.60 -1.425741 2.466908

Degrees-of-freedom method: satterthwaite
Confidence level used: 0.95

$contrasts
 contrast            estimate        SE   df t.ratio p.value
 M8-0540 - S5-0395 0.1352213 0.3507697 6.99   0.385  0.7113


>   plot(resid(lme3)~predict(lme3))
```

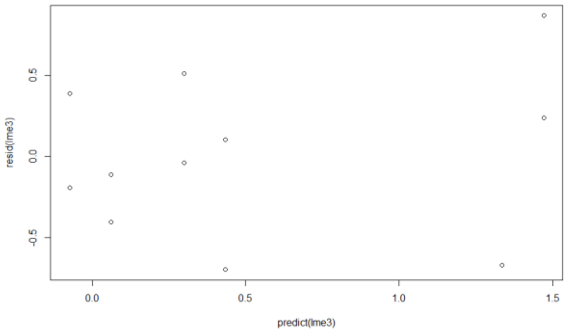


```
>   hist(resid(lme3))
```

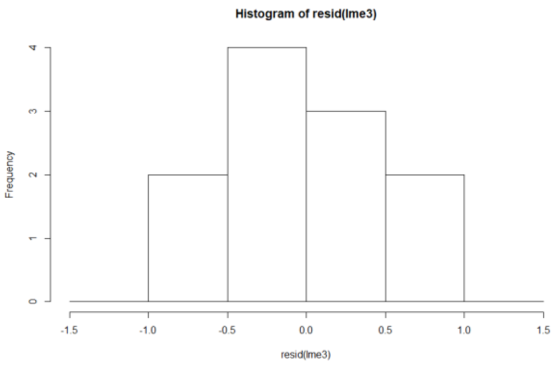


```
>   ls.m1 = lsmeans(lme3, pairwise~Strain)
>   cld(ls.m1)
 Strain     lsmean        SE   df  lower.CL upper.CL .group
 S5-0395 0.5205833 0.5222783 2.60 -1.425741 2.466908  1
 M8-0540 0.6558046 0.5093658 2.36 -1.242400 2.554009  1

Degrees-of-freedom method: satterthwaite
Confidence level used: 0.95
significance level used: alpha = 0.05
>   qqnorm(resid(lme3))
>   qqline(resid(lme3))
```

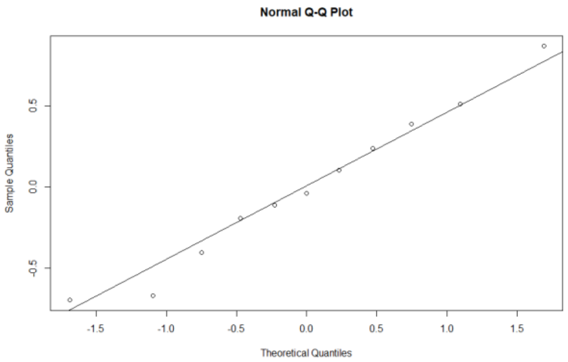


```
>   summary(ls.m1)
$lsmeans
 Strain     lsmean        SE   df  lower.CL upper.CL
 M8-0540 0.6558046 0.5093658 2.36 -1.242400 2.554009
 S5-0395 0.5205833 0.5222783 2.60 -1.425741 2.466908

Degrees-of-freedom method: satterthwaite
Confidence level used: 0.95

$contrasts
 contrast            estimate        SE   df t.ratio p.value
 M8-0540 - S5-0395 0.1352213 0.3507697 6.99   0.385  0.7113
```
